# Supplementary material for: impHFrEF trial: study protocol for an open-label, multicentre study of improvement the outcome of patients with heart failure in China using a mobile hEalth-supported platForm
Source: BMJ Open. 2024 Mar 29;14(3):e081011. doi: 10.1136/bmjopen-2023-081011 (PMC10982805; doi:10.1136/bmjopen-2023-081011)
Supplement: Author's manuscript [file bmjopen-2023-081011.draft_revisions.pdf]

BMJ Open is committed to open peer review. As part of this commitment we make the peer review history of every article we publish publicly available.

When an article is published we post the peer reviewers' comments and the authors' responses online. We also post the versions of the paper that were used during peer review. These are the versions that the peer review comments apply to.

The versions of the paper that follow are the versions that were submitted during the peer review process. They are not the versions of record or the final published versions. They should not be cited or distributed as the published version of this manuscript.

BMJ Open is an open access journal and the full, final, typeset and author-corrected version of record of the manuscript is available on our site with no access controls, subscription charges or pay-per-view fees (http://bmjopen.bmj.com).

If you have any questions on BMJ Open's open peer review process please email info.bmjopen@bmj.com

BMJ Open

The impHFrEF trial: study protocol for an open-label, multi-center study of improvement the outcome of patients with Heart Failure using a mobile hEalth-supported platForm

Journal:	*BMJ Open*
Manuscript ID	bmjopen-2023-081011
Article Type:	Protocol
Date Submitted by the Author:	17-Oct-2023
Complete List of Authors:	Chen, Fuzhong; Jiangsu Province Hospital and Nanjing Medical University First Affiliated Hospital, Department of Cardiology<br>Li, Guangjuan; Nanjing Medical University, Department of Cardiology; the Friendship Hospital of Yili Kazak Autonomous Prefecture, Nanjing Medical University<br>Zhang, Xinxin; Northern Jiangsu People's Hospital, Department of Cardiology<br>Shen, Qin; Jiangsu Province Hospital and Nanjing Medical University First Affiliated Hospital, Department of Cardiology<br>Wang, Fangfang; Changzhou No.2 People's Hospital, Department of Cardiology<br>Dong, Xiaoyu; Jiangsu Province Hospital and Nanjing Medical University First Affiliated Hospital, Department of Cardiology<br>Zou, Yu; Jiangsu Province Hospital and Nanjing Medical University First Affiliated Hospital, Department of Cardiology<br>Chen, Wensen; Jiangsu Province Hospital and Nanjing Medical University First Affiliated Hospital, Office of Infection Management<br>Xu, Bing; Northern Jiangsu People's Hospital, Department of Cardiology<br>Wang, Junhong; Jiangsu Province Hospital and Nanjing Medical University First Affiliated Hospital, Department of Cardiology; Jiangsu health administration and development research center
Keywords:	Clinical Trial, Heart failure < CARDIOLOGY, Prognosis

SCHOLARONE™
Manuscripts

The impHFrEF trial: study protocol for an open-label, multi-center study of **imp**rovement the outcome of patients with Heart Failu**r**e using a mobile hEalth-supported platForm

Author: Chen Fuzhong[1#], Li Guangjuan[2,3#], Zhang Xinxin[4#], Shen Qin[1], Wang Fangfang[5], Dong Xiaoyu[1], Zou Yu[1], Chen Wensen[6], Xu Bing [4*], Wang Junhong[1,7*]

[1]Department of Cardiology, the First Affiliated Hospital of Nanjing Medical University, Nanjing, China.

[2]Nanjing Medical University

[3]Department of Cardiology, the Friendship Hospital of Yili Kazak Autonomous Prefecture, Nanjing Medical University, ILY, China.

[4]Department of Cardiology, Northern Jiangsu People's Hospital, Yangzhou, China.

[5]Department of Cardiology, Changzhou No.2 People's Hospital, Changzhou, China.

[6]Office of Infection Management, the First Affiliated Hospital of Nanjing Medical University, Nanjing, China.

[7]Jiangsu health administration and development research center, Nanjing, China.

#These authors contributed equally.

* Corresponding to Dr. Xu Bing, Email: nyxb1980@163.com and Prof. Wang Junhong, E-mail: wangjunhong@jsph.org.cn

For peer review only

Abstract

Background

Patients with chronic heart failure (CHF) often have a long duration of illness, difficulty in attending follow-up visits, and poor adherence to treatment. As a result, they frequently cannot receive guideline-directed medical therapy (GDMT) at the desired or maximum tolerable drug dosage. This leads to high hospitalization and mortality rates for heart failure (HF) patients. Therefore, effective management and monitoring of patients with heart failure to ensure they receive GDMT is crucial for improving their prognosis.

Design and methods

This is a multicenter, open-label, randomized, parallel-group study involving patients with CHF across five centers. The study aims to assess the impact of an optimized guideline-directed medical therapy (GDMT) model for heart failure patients, established on a mobile health (mHealth) platform, compared to a control group. The primary composite outcome is worsening heart failure events (hospitalization or emergency treatment with intravenous fluids) or cardiovascular death. Patients must have a left ventricular ejection fraction (LVEF) of less than 50% and be receiving medication titration therapy that has not yet reached the target dose, with a modest increase in N-terminal pro-B-type natriuretic peptide level. The primary endpoint is a composite of cardiogenic death and hospitalization for worsening heart failure

For peer review only

symptoms.

Conclusions

The study aims to establish an optimal management model for patients with heart failure based on a mobile health (mHealth) platform. The expectation is that this model will provide an improved prognosis for patients with chronic heart failure and save social healthcare resources.

Keywords

Clinical trial • Heart failure • Optimized management model • moblile health(mHealth) platform

Introduction

The term chronic heart failure (CHF) refers to a complex clinical syndrome resulting from abnormal changes in the structure and/or function of the heart owning to numerous factors, leading to impaired ventricular systolic and/or diastolic function[1 2]. The main manifestations are dyspnea, fatigue, and fluid retention (pulmonary circulation congestion, systemic circulation congestion, and peripheral edema) [1 3]. Most patients with acute heart failure (AHF) have partial remission of symptoms after hospitalization and are referred to chronic heart failure; Patients with chronic heart failure often require hospitalization due to acute exacerbation of various triggers. Heart failure has become an important public health issue worldwide due to its high incidence, rehospitalization

For peer review only

rate, and death rate.[4 5] China-HF study shows a 4.1% mortality rate for hospitalized patients with heart failure in China[5]. The 2012-2015 Chinese Epidemiological Survey showed that the prevalence of heart failure among adults over 35 years old in China was 1.3% (estimated 13.7 million)[6]. As China's population ages and the incidence of chronic diseases such as coronary heart disease, hypertension, and diabetes increases, improvements in medical care have led to longer survival periods for patients with heart disease, resulting in a continuing trend of increased heart failure prevalence in China.

Guideline-directed medical therapy (GDMT) is a cornerstone of care for patients with CHF. In terms of drug therapy, angiotensin-converting enzyme inhibitors (ACEIs) or angiotensin II receptor antagonists (ARBs), beta-blockers (BBs), and mineralocorticoid receptor antagonists (MRAs) constitute the standard therapy for the treatment of chronic heart failure. This can significantly improve the clinical symptoms of patients with chronic heart failure and reduce the rate of rehospitalization and mortality. Over the past few years, a variety of new heart failure therapeutic agents have emerged that can improve prognosis. Among them, extensive evidence of the clinical benefit of angiotensin receptor-neprilysin inhibitors (ARNIs) and sodium-glucose co-transporter-2 inhibitors (SGLT2is) has led to a shift from the "ACEI/ARB+BB+MRA" regimen to the " ARNI/ACEI/ARB+SGLT2i+BB+MRA " regimen for improving the prognosis of patients with heart failure with reduced ejection fraction [3 7-9].

Clinical guidelines emphasize the importance of achieving the target dose of guideline-

For peer review only

guided medication therapy (GDMT) as much as possible[3 7]. However, due to the long duration of heart failure, it is difficult for patients to be rechecked and examined after discharge due to medical and economic constraints. In addition, severe side effects such as sinus bradycardia, atrioventricular block, and blood pressure reduction can occur during β-blockers, ACEIs/ARBs, and ARNIs therapy, leading to exacerbation and recurrence of heart failure. As a result, patients' adherence to treatment is poor after discharge from the hospital, making it impossible for patients to receive the target dose of medication. This ultimately leads to high rates of rehospitalization and death from heart failure. Recent studies have shown that even in the medically advanced United States, the vast majority of patients with heart failure are not titrated to the target dose or the maximum tolerated dose of BBs and ACEIs/ARBs[10-12]. Therefore, effective follow-up and management of patients are key to improving their prognosis of patients with heart failure and saving healthcare resources.

Recent studies in China and abroad have attempted to use existing social media tools such as WeChat, Facebook, and Twitter to manage patients with coronary heart disease (CHD) and other cardiovascular diseases to effectively reduce the incidence of cardiovascular events in patients with coronary heart disease. Ge and his team reported in Lancet on the successful use of a WeChat social platform to guide the treatment of patients with CHD through refined management for rehabilitation and secondary prevention[13]. As a result, an APP has been developed for patients with chronic heart failure based on the mobile health(mHealth) platform. The APP can be used to monitor

For peer review only

the daily condition of the patient, allowing the physician and patient to communicate in real-time through the software. This establishes a remote monitoring platform with the participation of doctors and patients. By monitoring the arrhythmias of patients with heart failure and their responsiveness to medication, patients can be effectively monitored remotely and guided in the use of medication. Ultimately, patients with heart failure will be effectively managed in a timely and scientific manner, reducing the number of repeated hospitalizations, lowering the death rate of HF patients, and reducing the medical burden on society.

Consequently, the implementation of this study can establish an optimal guideline-directed medical therapy (GDMT) management model for patients with heart failure based on the mHealth platform. This model can be effectively promoted and popularized, ultimately improving the prognosis of patients with HF and saving social medical resources.

Methods

The study is a multicenter, parallel-group, randomized, open-label, prospective trial in patients with chronic heart failure. The study was registered in the Chinese Clinical Trial Registry on 2022-02-07. The registration number of the study is ChiCTR2200056527.

For peer review only

Study design and conduct

Trial Design Overview

The study is a multicenter, prospective, open-label study. A total of 5 centers participated in this study: Jiangsu Provincial People's Hospital, Northern Jiangsu People's Hospital, the Second Affiliated Hospital of Nanjing Medical University, Changzhou No.2 People's Hospital, and the Friendship Hospital of ILY Kazak Autonomous Prefecture. A total of 680 patients with CHF are planned to be randomly enrolled and assigned 1:1 to the mhealth optimized GDMT group (mGDMT group) and the control group, with 340 patients in each group. The population included is heart failure medication not titrated to the target doses or maximum tolerated doses. Patients in the mGDMT group are managed individually and precisely through a mHealth platform application (App), and the dosage of beta-blockers and other HF treatment medications are titrated and adjusted to the patient's tolerated target dose according to the results of the remote mHealth platform. The control group receives the current clinical routine follow-up and treatment. Follow-up will be performed at the intervals of 1 month, 3 months, 6 months, and 12 months after enrollment for a total of 12 months to compare clinical symptoms, cardiac function classification, incidence of adverse events, hospitalization rate, mean number of days in hospital, and mortality between the two groups. Figure 1 illustrates the trial procedures of the study.

For peer review only

The mHealth platform App construction

The mHealth platform App builds an interactive platform for doctors and patients to participate together. The App includes a doctor-side and a patient-side portal. The patient side of the software requires the patient to bind to the appropriate doctor and clock in daily on their weight, urine output, blood pressure, heart rates, heart failure symptoms, and other indicators (laboratory results or examination for local review). The patient-side data is uploaded and presented in a visualized way on the doctor-side. The physician understands the patients' medication reaction based on the daily punch card data and communicates effectively with the patient through the App platform dialogue tool. The corresponding physician makes precise and standardized adjustments to the heart failure medication and treatment according to the patients' condition. At the same time, the platform sets up an alarm function for extreme values to alert physicians when abnormal values of key indicators appear in patients, facilitating timely adjustment of patients' medication regimens. Throughout the visit, the importance of patient adherence with program requirements regarding daily measurements and symptom reporting is emphasized to ensure timely identification of potential changes in their condition. Figure 2 demonstrates the model for optimal management of patients with heart failure based on a mHealth App.

Patients

The population included in this study is patients with heart failure who have new or ongoing heart failure medication that has not yet reached target doses and requires

For peer review only

adjustment of medication to target or goal measures, or patients with heart failure who have been treated to stop the intravenous vasoactive medication and then switched to oral medication after stabilization. The patient's left ventricular ejection fraction (LVEF) as measured by the Simpson method needs to be <50% and the New York Heart Association (NYHA) Classes II-IV (NYHA Classes IV in stable phase). The patient's brain natriuretic peptide (BNP) concentration should be ≥150 pg/ml or N-terminal pro-B-type natriuretic peptide (NT-proBNP) ≥600 pg/ml, or BNP ≥100 pg/ml if the patient has been hospitalized for heart failure within 12 months (NT-proBNP ≥400 pg/ml). Subjects must meet the age limit of 18 years, be proficient in the use of electronic devices (Proficiency in using WeChat app), and be willing to provide written informed consent.

The key exclusion criteria for the impHFrEF trial include Somatic Activity Disorders and Psychiatric Disorders, Chronic obstructive pulmonary disease (COPD) and Asthma, Malignant tumor patients with a life expectancy of less than 1 year, Plans to receive device or surgical treatment such as a heart transplant, left ventricular assist device, CRTD implantation or valve replacement surgery during the follow-up period, Recurrent episodes of intractable heart failure, Various correctable secondary causes of heart failure including hyperthyroidism, anemic heart disease, congenital heart disease, organic valvular heart disease. Patients with renal failure (creatinine clearance <30 ml/min) or on dialysis treatment, hyperkalemia (blood potassium >5.5 mmol/l) at the time of enrollment and those who are participating in other clinical trials are also

For peer review only

excluded.

Randomization

Once patients meet the criteria for randomization, they are assigned by investigators through a central randomization system. Patients in the study are centrally randomized and competitively enrolled in either the mGDMT group or the normal control groups.

Study visits and follow-up

Patients who meet the inclusion criteria will be evaluated upon admission and randomly assigned to either the mGDMT group or the control group after signing an informed consent form. Baseline information, including clinical examination and laboratory measurements, will be collected. Follow-up visits will be conducted on days 30 ± 3, 90 ± 3, 180 ± 3, and 360 ± 3 after randomization. These visits will focus on heart failure symptoms, signs, volume status, and adverse events. ECG, echocardiography, and laboratory results such as troponin, pro-BNP, liver and kidney function will also be reviewed. The schedule for data acquisition is shown in Table 1.

Outcomes

The primary endpoint is a composite endpoint consisting of cardiac death and hospitalization due to worsening heart failure symptoms. Worsening heart failure symptoms are defined as the presence of heart failure that cannot be controlled by oral medications and requires hospitalization or emergency room treatment with intravenous medications.

For peer review only

The secondary endpoints primarily include：(i) all-cause death; (ii) cumulative heart failure admissions during the trial; (iii) changes in the 6-minute walk test and heart failure biomarkers; (iv) changes from baseline to 12 months in the total symptom score using the Kansas City Cardiomyopathy Questionnaire (KCCQ)[14]; (v) changes in Self-Rating Anxiety Scale (SAS) scores during the visits[15].

The study is expected to collect as much clinical information as possible from patients during each visit. This information will be used to compare clinical symptoms, cardiac function class, incidence of adverse events, hospitalization rate, mean days in the hospital, and mortality between the two groups of patients. The efficacy of heart failure medication and adverse events will be monitored and recorded.

Statistical considerations

Sample size assumptions

The incidence rate of endpoint events in the control group was determined based on previous research findings. In the PARADIM-HF study, the LCZ696 group demonstrated an incidence rate of 21% during a follow-up period of 27 months[16]. Given the recent advances in pharmacological and device-based treatments for heart failure, we assume an incidence rate of 15% (data 2019 for HFrEF patients, the first affiliated hospital with Nanjing Medical University) for the control group. It has been reported that remote monitoring can lead to a decrease in mortality rates [0.36 to 0.80] and

For peer review only

hospitalization rates [0.39 to 0.95] associated with heart failure when compared to the control group[17]. Accordingly, we postulate that the incidence rate of events in the strict management group could be effectively reduced to approximately 50% (equating to an incidence rate of 8%). With a test efficacy of 0.8 and a bilateral alpha of 0.05, a sample size ratio of 1:1 for inclusion of the control and mGDMT groups, and a lost-to-review rate of 5%, a minimum sample size of 340 cases is required for each of the two groups. Therefore, the study expects to include a total of 680 patients.

Methods for statistical analyses

The statistical methods to be used in the experiment have been preplanned. R (version 4.2.2) and SPSS (version 27) will be used for all analyses. The primary and secondary efficacy analyses will be performed on the full analysis set, according to the principle of intention-to-treat. The full analysis set includes all randomized patients who have consented and started receiving the indicated treatment.

Data conforming to a normal distribution will be expressed as mean ± standard deviation and compared using an independent samples t-test. Variables with a skewed distribution will be expressed as the median and interquartile range (IQR) and compared using a nonparametric test. Counts and percentages will be calculated for categorical data and evaluated using Chi-square or Fisher exact tests. The Kaplan-Meier (K-M) method will be used to assess the difference in the incidence of endpoint events between the two groups of patients.

For peer review only

Ancillary studies

After obtaining informed consent from patients, blood samples will be collected at baseline and during follow-up for the measurement of biomarkers of interest.

Trial design and governance

The study was designed by the Cardiovascular Medicine Department of the First Affiliated Hospital of Nanjing Medical University and reviewed by its Ethics Committee. The study has completed clinical trial registration and obtained a registration number. There are 5 study centers, and the study will be supervised by the ethics committees of their respective centers during the study implementation.

Discussion

Several aspects of the design of this study merit discussion.

Key aspects of trial design

One of the most challenging issues in managing heart failure is the high readmission rate and death rate due to worsening heart failure symptoms. Effective pharmacologic therapy can improve patients' clinical symptoms and reduce readmission and mortality rates. However, patients often lack effective management after being discharged from the hospital, and most fail to achieve the target doses (or maximum tolerated doses) of drug therapy[5 10-12]. Telemanagement is one of the most important components of managing patients with CHF. In some previous trials of remotely titrated BBs, a

For peer review only

significantly higher percentage of patients in the remote titration group achieved their target dose or did so in a relatively shorter time [18-20]. Previous studies have shown that patients with heart failure can benefit from a remote patient management approach[21-23]. These studies provide various forms of remote monitoring interventions for HF patients, which can improve their clinical prognosis and reduce mortality and hospitalization rates. These interventions can supplement routine care, enhance patients' self-management abilities, and improve their quality of life. Therefore, the study establishes an optimal management model for patients with heart failure based on a mHealth platform APP to provide medication guidance (remote titration of HF medication) and remote management for patients with HF. Ultimately, the research hopes to reduce patients' hospitalization and death rates and alleviate their symptoms.

The shift in heart failure drug regimen

In recent years, a variety of drugs have emerged that improve the prognosis of patients with heart failure. The substantial evidence of the clinical benefit of ARNIs and SGLT2is has already contributed to a transformation in treatment options for patients with heart failure with reduced ejection fraction[16 24-26]. Therefore, according to the guideline recommendations, the study uses the " ARNIs/ACEIs/ARBs+ SGLT2is+ BBs+ MRAs" regimen as the base drug therapy for heart failure [3 7].

Key inclusion and exclusion criteria

The population included in the study primarily consists of patients with heart failure

For peer review only - http://bmjopen.bmj.com/site/about/guidelines.xhtml

who are receiving heart failure medication that has not yet reached their target values and need to adjust their drug therapy to the target dose. With our optimized management model and adjusted drug therapy, the study expects a more significant reduction in hospitalization rates, hospitalization days, and mortality in this group of patients. Patients who are unable to receive standard heart failure medication, such as those with chronic obstructive pulmonary disease, asthma, or hyperkalemia (blood potassium >5.5 mmol/l at enrollment), are excluded from the study. Correctable secondary causes of cardiac insufficiency, including hyperthyroid heart disease and anemic heart disease, are also excluded from the study.

Safety considerations

Patients with heart failure often have a changing condition, with mortality and rehospitalization rates as high as 15% and 30% within 2-3 months of their discharge from the hospital[27]. Therefore, investigators need to evaluate patients' uploaded heart rates, blood pressure, heart failure symptoms, and test results to adjust therapeutic drug doses according to their hemodynamic status and fluid retention. If patients develop renal insufficiency or hyperkalemia during treatment, the medication should also be adjusted immediately according to the guidelines[3]. The trial should be terminated if there is a rapid deterioration in renal function (50% increase in creatinine level or 0.3 mg/dl increase in absolute creatinine level compared to 48 hours before) or severe sinus bradycardia requiring pacing therapy.

For peer review only - http://bmjopen.bmj.com/site/about/guidelines.xhtml

Current status

The First Affiliated Hospital of Nanjing Medical University ethically cleared the study on December 22, 2021, and clinical trial registration was completed on February 7, 2022. Ethics number is 2021-SR-530. The mHealth platform APP was constructed on June 1, 2022. The first subject was recruited and randomized to groups on June 29, 2022. A later phase will rapidly recruit subjects and complete randomization.

Conclusions

Effective pharmacotherapy is key to the treatment of patients with chronic heart failure. However, owing to the long duration of heart failure, the difficulty for patients to follow up (limited by medical level and economic conditions), and the fear of drug side effects, patients' treatment adherence is poor. As a result, most patients do not reach the target dose (or the maximum tolerated dose), leading to a high hospitalization rate and mortality in patients with HF. The trial expects that remote titration, strict follow-up, and remote management of patients will reduce hospitalization and mortality rates and improve the quality of life for patients with heart failure. The study's long-term goal is to establish an optimal management model for patients with heart failure based on a mHealth platform, which will ultimately improve the prognosis of patients with heart failure and save social healthcare resources.

For peer review only

Supplementary Information

Funding

This work was supported by the National Natural Science Foundation of China (NSFC 82170269), Jiangsu Province Hospital (the first affiliated hospital with Nanjing Medical University) clinical capacity enhancement project (JSPH-MB-2021-15), the Foundation of Clinical Medical Research Center of Yili Autonomous Prefecture (YL2020ms09), and the open project of Jiangsu health administration and development research center (JSHD2022009).

References

1. Guan X, Sun Z. The Role of Intestinal Flora and Its Metabolites in Heart Failure. *Infection and drug resistance* 2023;16:51-64. doi: 10.2147/idr.S390582 [published Online First: 2023/01/14]

2. Kanodia J, Lo A, Baldwin RM, et al. Safety, Pharmacokinetics, and Pharmacodynamics of TD-0714, a Novel Potent Neprilysin Inhibitor in Healthy Adult and Elderly Subjects. *Clinical and translational science* 2020;13(6):1307-15. doi: 10.1111/cts.12831 [published Online First: 2020/06/09]

3. McDonagh TA, Metra M, Adamo M, et al. 2021 ESC Guidelines for the diagnosis and treatment of acute and chronic heart failure. *European heart journal* 2021;42(36):3599-726. doi: 10.1093/eurheartj/ehab368 [published Online First: 2021/08/28]

4. Chow SL, Maisel AS, Anand I, et al. Role of Biomarkers for the Prevention, Assessment, and

For peer review only

Management of Heart Failure: A Scientific Statement From the American Heart Association. *Circulation* 2017;135(22):e1054-e91. doi: 10.1161/cir.0000000000000490 [published Online First: 2017/04/28]

5. Zhang Y, Zhang J, Butler J, et al. Contemporary Epidemiology, Management, and Outcomes of Patients Hospitalized for Heart Failure in China: Results From the China Heart Failure (China-HF) Registry. *Journal of cardiac failure* 2017;23(12):868-75. doi: 10.1016/j.cardfail.2017.09.014 [published Online First: 2017/10/17]
6. Hao G, Wang X, Chen Z, et al. Prevalence of heart failure and left ventricular dysfunction in China: the China Hypertension Survey, 2012-2015. *European journal of heart failure* 2019;21(11):1329-37. doi: 10.1002/ejhf.1629 [published Online First: 2019/11/21]
7. Heidenreich PA, Bozkurt B, Aguilar D, et al. 2022 AHA/ACC/HFSA Guideline for the Management of Heart Failure: Executive Summary: A Report of the American College of Cardiology/American Heart Association Joint Committee on Clinical Practice Guidelines. *Circulation* 2022;145(18):e876-e94. doi: 10.1161/cir.0000000000001062 [published Online First: 2022/04/02]
8. Maddox TM, Januzzi JL, Jr., Allen LA, et al. 2021 Update to the 2017 ACC Expert Consensus Decision Pathway for Optimization of Heart Failure Treatment: Answers to 10 Pivotal Issues About Heart Failure With Reduced Ejection Fraction: A Report of the American College of Cardiology Solution Set Oversight Committee. *Journal of the American College of Cardiology* 2021;77(6):772-810. doi: 10.1016/j.jacc.2020.11.022 [published Online First: 2021/01/16]
9. McMurray JJV, Packer M. How Should We Sequence the Treatments for Heart Failure and

For peer review only

a Reduced Ejection Fraction?: A Redefinition of Evidence-Based Medicine. *Circulation* 2021;143(9):875-77. doi: 10.1161/circulationaha.120.052926 [published Online First: 2020/12/31]

10. Chang HY, Wang CC, Wei J, et al. Gap between guidelines and clinical practice in heart failure with reduced ejection fraction: Results from TSOC-HFrEF registry. *Journal of the Chinese Medical Association : JCMA* 2017;80(12):750-57. doi: 10.1016/j.jcma.2017.04.011 [published Online First: 2017/10/17]
11. Greene SJ, Fonarow GC, DeVore AD, et al. Titration of Medical Therapy for Heart Failure With Reduced Ejection Fraction. *Journal of the American College of Cardiology* 2019;73(19):2365-83. doi: 10.1016/j.jacc.2019.02.015 [published Online First: 2019/03/08]
12. Jungbauer CG, Maier LS, Emoto K, et al. Achieving Guideline-Directed Heart Rate ControlEarly Posthospitalization. *The American journal of cardiology* 2019;123(7):1096-100. doi: 10.1016/j.amjcard.2018.12.032 [published Online First: 2019/01/27]
13. Dorje T, Zhao G, Tso K, et al. Smartphone and social media-based cardiac rehabilitation and secondary prevention in China (SMART-CR/SP): a parallel-group, single-blind, randomised controlled trial. *The Lancet Digital health* 2019;1(7):e363-e74. doi: 10.1016/s2589-7500(19)30151-7 [published Online First: 2020/12/17]
14. Green CP, Porter CB, Bresnahan DR, et al. Development and evaluation of the Kansas City Cardiomyopathy Questionnaire: a new health status measure for heart failure. *Journal of the American College of Cardiology* 2000;35(5):1245-55. doi:

For peer review only

10.1016/s0735-1097(00)00531-3 [published Online First: 2000/04/12]

15. Zung WW. The measurement of affects: depression and anxiety. *Modern problems of pharmacopsychiatry* 1974;7(0):170-88. doi: 10.1159/000395075 [published Online First: 1974/01/01]

16. McMurray JJ, Packer M, Desai AS, et al. Angiotensin-neprilysin inhibition versus enalapril in heart failure. *The New England journal of medicine* 2014;371(11):993-1004. doi: 10.1056/NEJMoa1409077 [published Online First: 2014/09/02]

17. Kotb A, Cameron C, Hsieh S, et al. Comparative effectiveness of different forms of telemedicine for individuals with heart failure (HF): a systematic review and network meta-analysis. *PloS one* 2015;10(2):e0118681. doi: 10.1371/journal.pone.0118681 [published Online First: 2015/02/26]

18. D'Onofrio A, Palmisano P, Rapacciuolo A, et al. Effectiveness of a management program for outpatient clinic or remote titration of beta-blockers in CRT patients: The RESTORE study. *International journal of cardiology* 2017;236:290-95. doi: 10.1016/j.ijcard.2017.02.015 [published Online First: 2017/02/12]

19. Palmisano P, Ammendola E, D'Onofrio A, et al. Evaluation of synergistic effects of resynchronization therapy and a β-blocker up-titration strategy based on a predefined patient-management program: the RESTORE study. *Clinical cardiology* 2015;38(1):2-7. doi: 10.1002/clc.22352 [published Online First: 2015/01/13]

20. Spaeder J, Najjar SS, Gerstenblith G, et al. Rapid titration of carvedilol in patients with congestive heart failure: a randomized trial of automated telemedicine versus frequent outpatient clinic visits. *American heart journal* 2006;151(4):844.e1-10. doi:

For peer review only

10.1016/j.ahj.2005.06.044 [published Online First: 2006/03/30]

21. Cowie MR, Anker SD, Cleland JGF, et al. Improving care for patients with acute heart failure: before, during and after hospitalization. *ESC heart failure* 2014;1(2):110-45. doi: 10.1002/ehf2.12021 [published Online First: 2014/12/01]

22. Koehler F, Koehler K, Deckwart O, et al. Efficacy of telemedical interventional management in patients with heart failure (TIM-HF2): a randomised, controlled, parallel-group, unmasked trial. *Lancet (London, England)* 2018;392(10152):1047-57. doi: 10.1016/s0140-6736(18)31880-4 [published Online First: 2018/08/30]

23. Ong MK, Romano PS, Edgington S, et al. Effectiveness of Remote Patient Monitoring After Discharge of Hospitalized Patients With Heart Failure: The Better Effectiveness After Transition -- Heart Failure (BEAT-HF) Randomized Clinical Trial. *JAMA internal medicine* 2016;176(3):310-8. doi: 10.1001/jamainternmed.2015.7712 [published Online First: 2016/02/10]

24. Guthrie R. Canagliflozin and cardiovascular and renal events in type 2 diabetes. *Postgraduate medicine* 2018;130(2):149-53. doi: 10.1080/00325481.2018.1423852 [published Online First: 2018/01/04]

25. McMurray JJV, Solomon SD, Inzucchi SE, et al. Dapagliflozin in Patients with Heart Failure and Reduced Ejection Fraction. *The New England journal of medicine* 2019;381(21):1995-2008. doi: 10.1056/NEJMoa1911303 [published Online First: 2019/09/20]

26. Zinman B, Wanner C, Lachin JM, et al. Empagliflozin, Cardiovascular Outcomes, and Mortality in Type 2 Diabetes. *The New England journal of medicine* 2015;373(22):2117-

For peer review only

28. doi: 10.1056/NEJMoa1504720 [published Online First: 2015/09/18]

27. Greene SJ, Fonarow GC, Vaduganathan M, et al. The vulnerable phase after hospitalization for heart failure. *Nature reviews Cardiology* 2015;12(4):220-9. doi: 10.1038/nrcardio.2015.14 [published Online First: 2015/02/11]

For peer review only

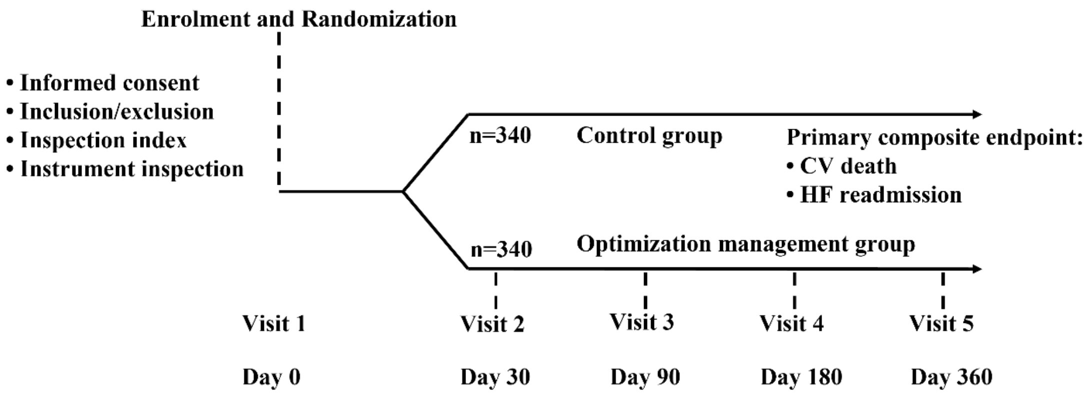

Figure 1 Trial design. CV, cardiovascular; HF, heart failure.

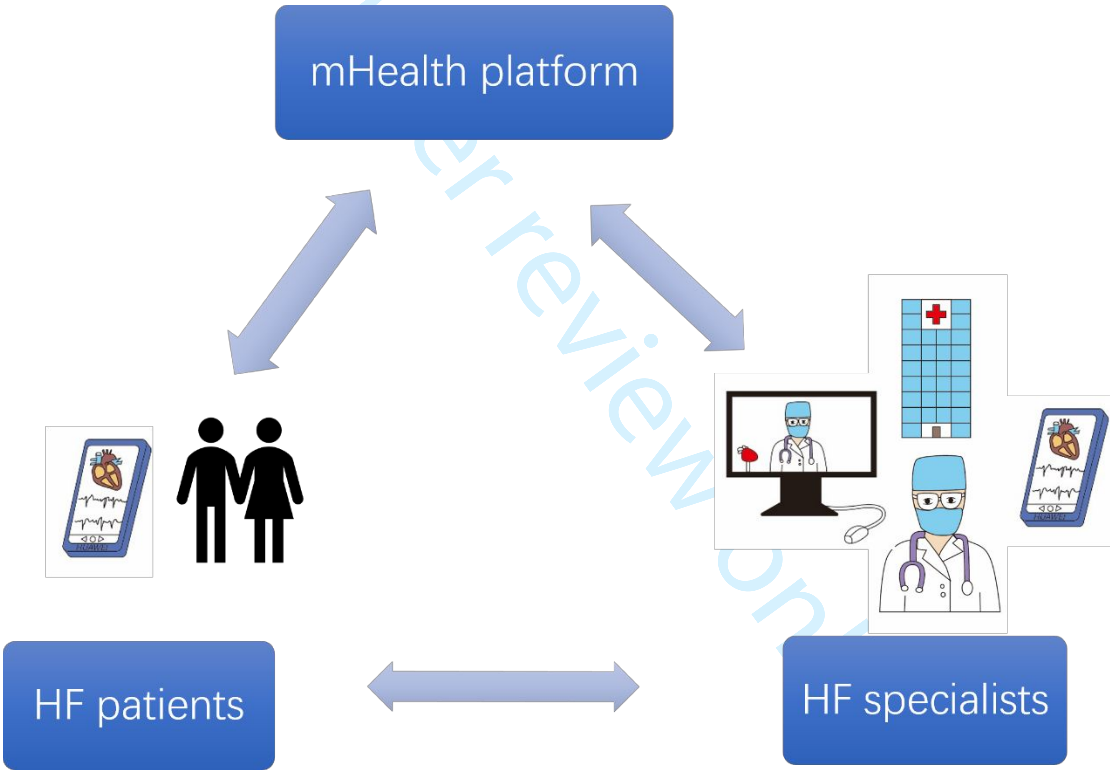

Figure 2 Optimized GDMT model for managing HF patients based on the mHealth platform. mHealth, mobile health; HF, heart failure; GDMT, guideline directed medical therapy.

For peer review only - http://bmjopen.bmj.com/site/about/guidelines.xhtml

Textbox 1. Patient inclusion criteria.

- Willing to provide written informed consent
- 18 years or older
- Left ventricular ejection fraction (LVEF) as measured by the Simpson method needs to be <50% and the New York Heart Association (NYHA) Classes II-IV (NYHA IV in stable phase)
- The patient's brain natriuretic peptide (BNP) concentration should be ≥150 pg/ml or N-terminal pro-B-type natriuretic peptide (NT-proBNP) ≥600 pg/ml, or BNP ≥100 pg/ml (NT-proBNP ≥400 pg/ml) if the patient has been hospitalized for heart failure within 12 months
- Heart failure patients with new or ongoing anti-heart failure medication that has not yet reached target doses and requires adjustment of medication to target or goal measures, or heart failure patients who have been treated to stop the intravenous vasoactive medication and then switched to oral medication after stabilization
- Patients should be proficient in the use of electronic devices

Textbox 2. Patient exclusion criteria.

- Somatic Activity Disorders and Psychiatric Disorders
- Chronic obstructive pulmonary disease (COPD) and Asthma
- Malignant tumor patients (life expectancy < 1 year)
- Plans to receive device or surgical treatment (such as a heart transplant, left ventricular assist device or CRTD implantation and valve replacement surgery during the follow-up period)
- Recurrent episodes of intractable heart failure, Various correctable secondary causes of heart failure (including hyperthyroidism, anemic heart disease, congenital heart disease, organic valvular heart disease)
- Patients with renal failure (creatinine clearance <30 ml/min) or on dialysis treatment
- Hyperkalemia (blood potassium >5.5 mmol/l) at randomization
- Those who are participating in other clinical trials

For peer review only

Table 1. Schedule for data acquisition indicated by checkmarks at the specified time point.

Data collected	Baseline	Day 30	Day 90	Day 180	Day 360
Demographics	✓	N/A[a]	N/A	N/A	N/A
History	✓	N/A	N/A	N/A	N/A
Vital signs	✓	✓	✓	✓	✓
Signs and symptoms	✓	✓	✓	✓	✓
NYHA[b] class	✓	✓	✓	✓	✓
6-min walk test	✓	✓	✓	✓	✓
KCCQ[c] scores	✓	✓	✓	✓	✓
Laboratory results					
BNP[d] levels	✓			✓	✓
Troponin	✓			✓	✓
Renal function	✓			✓	✓
Liver function	✓			✓	✓
Examination results					
ECG[e]	✓			✓	✓
Echocardiography	✓			✓	✓
Endpoints					
Cardiac death	N/A	✓	✓	✓	✓
Number of HF[f]-related hospitalizations since enrollment	N/A	✓	✓	✓	✓
Number of days in the hospital since enrollment	N/A	✓	✓	✓	✓
All-cause death	N/A	✓	✓	✓	✓

[a]N/A: not applicable.

For peer review only

[b]NYHA: New York Heart Association.

[c]KCCQ: Kansas City Cardiomyopathy Questionnaire.

[d]BNP: brain natriuretic peptide.

[e]ECG: Electrocardiogram.

[f]HF: heart failure.

For peer review only

Reporting checklist for protocol of a clinical trial.

Based on the SPIRIT guidelines.

Instructions to authors

Complete this checklist by entering the page numbers from your manuscript where readers will find each of the items listed below.

Your article may not currently address all the items on the checklist. Please modify your text to include the missing information. If you are certain that an item does not apply, please write "n/a" and provide a short explanation.

Upload your completed checklist as an extra file when you submit to a journal.

In your methods section, say that you used the SPIRITreporting guidelines, and cite them as:

Chan A-W, Tetzlaff JM, Gøtzsche PC, Altman DG, Mann H, Berlin J, Dickersin K, Hróbjartsson A, Schulz KF, Parulekar WR, Krleža-Jerić K, Laupacis A, Moher D. SPIRIT 2013 Explanation and Elaboration: Guidance for protocols of clinical trials. BMJ. 2013;346:e7586

		Reporting Item	Page Number
**Administrative information**			
Title	#1	Descriptive title identifying the study design, population, interventions, and, if applicable, trial acronym	1

For peer review only

Trial registration	#2a	Trial identifier and registry name. If not yet registered, name of intended registry	6
Trial registration: data set	#2b	All items from the World Health Organization Trial Registration Data Set	6
Protocol version	#3	Date and version identifier	n/a
Funding	#4	Sources and types of financial, material, and other support	16
Roles and responsibilities: contributorship	#5a	Names, affiliations, and roles of protocol contributors	1
Roles and responsibilities: sponsor contact information	#5b	Name and contact information for the trial sponsor	1,17
Roles and responsibilities: sponsor and funder	#5c	Role of study sponsor and funders, if any, in study design; collection, management, analysis, and interpretation of data; writing of the report; and the decision to submit the report for publication, including whether they will have ultimate authority over any of these activities	1,17
Roles and responsibilities: committees	#5d	Composition, roles, and responsibilities of the coordinating centre, steering committee, endpoint adjudication committee, data management team, and	13

For peer review only

		other individuals or groups overseeing the trial, if applicable (see Item 21a for data monitoring committee)	
**Introduction**			
Background and rationale	#6a	Description of research question and justification for undertaking the trial, including summary of relevant studies (published and unpublished) examining benefits and harms for each intervention	5,6
Background and rationale: choice of comparators	#6b	Explanation for choice of comparators	4-6
Objectives	#7	Specific objectives or hypotheses	6
Trial design	#8	Description of trial design including type of trial (eg, parallel group, crossover, factorial, single group), allocation ratio, and framework (eg, superiority, equivalence, non-inferiority, exploratory)	7
**Methods: Participants, interventions, and outcomes**			
Study setting	#9	Description of study settings (eg, community clinic, academic hospital) and list of countries where data will be collected. Reference to where list of study sites can be obtained	7

For peer review only

Eligibility criteria	#10	Inclusion and exclusion criteria for participants. If applicable, eligibility criteria for study centres and individuals who will perform the interventions (eg, surgeons, psychotherapists)	8、9
Interventions: description	#11a	Interventions for each group with sufficient detail to allow replication, including how and when they will be administered	7、8
Interventions: modifications	#11b	Criteria for discontinuing or modifying allocated interventions for a given trial participant (eg, drug dose change in response to harms, participant request, or improving / worsening disease)	15
Interventions: adherance	#11c	Strategies to improve adherence to intervention protocols, and any procedures for monitoring adherence (eg, drug tablet return; laboratory tests)	n/a
Interventions: concomitant care	#11d	Relevant concomitant care and interventions that are permitted or prohibited during the trial	n/a
Outcomes	#12	Primary, secondary, and other outcomes, including the specific measurement variable (eg, systolic blood pressure), analysis metric (eg, change from baseline, final value, time to event), method of aggregation (eg, median, proportion), and time point for each outcome. Explanation of the clinical relevance of chosen efficacy and harm outcomes is strongly recommended	10、11

For peer review only

Participant timeline	#13	Time schedule of enrolment, interventions (including any run-ins and washouts), assessments, and visits for participants. A schematic diagram is highly recommended (see Figure)	10
Sample size	#14	Estimated number of participants needed to achieve study objectives and how it was determined, including clinical and statistical assumptions supporting any sample size calculations	11、12
Recruitment	#15	Strategies for achieving adequate participant enrolment to reach target sample size	15,16
**Methods: Assignment of interventions (for controlled trials)**			
Allocation: sequence generation	#16a	Method of generating the allocation sequence (eg, computer-generated random numbers), and list of any factors for stratification. To reduce predictability of a random sequence, details of any planned restriction (eg, blocking) should be provided in a separate document that is unavailable to those who enrol participants or assign interventions	10
Allocation concealment mechanism	#16b	Mechanism of implementing the allocation sequence (eg, central telephone; sequentially numbered, opaque, sealed envelopes), describing any steps to conceal the sequence until interventions are assigned	10

For peer review only

Allocation: implementation	#16c	Who will generate the allocation sequence, who will enrol participants, and who will assign participants to interventions	10
Blinding (masking)	#17a	Who will be blinded after assignment to interventions (eg, trial participants, care providers, outcome assessors, data analysts), and how	6
Blinding (masking): emergency unblinding	#17b	If blinded, circumstances under which unblinding is permissible, and procedure for revealing a participant's allocated intervention during the trial	n/a
**Methods: Data collection, management, and analysis**			
Data collection plan	#18a	Plans for assessment and collection of outcome, baseline, and other trial data, including any related processes to promote data quality (eg, duplicate measurements, training of assessors) and a description of study instruments (eg, questionnaires, laboratory tests) along with their reliability and validity, if known. Reference to where data collection forms can be found, if not in the protocol	10、11
Data collection plan: retention	#18b	Plans to promote participant retention and complete follow-up, including list of any outcome data to be	10、11

For peer review only

		collected for participants who discontinue or deviate from intervention protocols	
Data management	#19	Plans for data entry, coding, security, and storage, including any related processes to promote data quality (eg, double data entry; range checks for data values). Reference to where details of data management procedures can be found, if not in the protocol	10、11
Statistics: outcomes	#20a	Statistical methods for analysing primary and secondary outcomes. Reference to where other details of the statistical analysis plan can be found, if not in the protocol	11、12
Statistics: additional analyses	#20b	Methods for any additional analyses (eg, subgroup and adjusted analyses)	11、12
Statistics: analysis population and missing data	#20c	Definition of analysis population relating to protocol non-adherence (eg, as randomised analysis), and any statistical methods to handle missing data (eg, multiple imputation)	12,13

Methods: Monitoring

Data monitoring: formal committee	#21a	Composition of data monitoring committee (DMC); summary of its role and reporting structure; statement of whether it is independent from the sponsor and competing interests; and reference to where further details about its charter can be found, if not in the	13

For peer review only

		protocol. Alternatively, an explanation of why a DMC is not needed	
Data monitoring: interim analysis	#21b	Description of any interim analyses and stopping guidelines, including who will have access to these interim results and make the final decision to terminate the trial	13
Harms	#22	Plans for collecting, assessing, reporting, and managing solicited and spontaneously reported adverse events and other unintended effects of trial interventions or trial conduct	15
Auditing	#23	Frequency and procedures for auditing trial conduct, if any, and whether the process will be independent from investigators and the sponsor	n/a
**Ethics and dissemination**			
Research ethics approval	#24	Plans for seeking research ethics committee / institutional review board (REC / IRB) approval	15,16
Protocol amendments	#25	Plans for communicating important protocol modifications (eg, changes to eligibility criteria, outcomes, analyses) to relevant parties (eg, investigators, REC / IRBs, trial participants, trial registries, journals, regulators)	n/a

Consent or assent	#26a	Who will obtain informed consent or assent from potential trial participants or authorised surrogates, and how (see Item 32)	9
Consent or assent: ancillary studies	#26b	Additional consent provisions for collection and use of participant data and biological specimens in ancillary studies, if applicable	12
Confidentiality	#27	How personal information about potential and enrolled participants will be collected, shared, and maintained in order to protect confidentiality before, during, and after the trial	10
Declaration of interests	#28	Financial and other competing interests for principal investigators for the overall trial and each study site	17
Data access	#29	Statement of who will have access to the final trial dataset, and disclosure of contractual agreements that limit such access for investigators	n/a
Ancillary and post trial care	#30	Provisions, if any, for ancillary and post-trial care, and for compensation to those who suffer harm from trial participation	15
Dissemination policy: trial results	#31a	Plans for investigators and sponsor to communicate trial results to participants, healthcare professionals, the public, and other relevant groups (eg, via publication, reporting in results databases, or other data sharing arrangements), including any publication restrictions	n/a

For peer review only

Dissemination policy: authorship	#31b	Authorship eligibility guidelines and any intended use of professional writers	1
Dissemination policy: reproducible research	#31c	Plans, if any, for granting public access to the full protocol, participant-level dataset, and statistical code	n/a

Appendices

Informed consent materials	#32	Model consent form and other related documentation given to participants and authorised surrogates	12、13
Biological specimens	#33	Plans for collection, laboratory evaluation, and storage of biological specimens for genetic or molecular analysis in the current trial and for future use in ancillary studies, if applicable	12、13

None The SPIRIT Explanation and Elaboration paper is distributed under the terms of the Creative Commons Attribution License CC-BY-NC. This checklist can be completed online using https://www.goodreports.org/, a tool made by the EQUATOR Network in collaboration with Penelope.ai

For peer review only

BMJ Open

The impHFrEF trial: study protocol for an open-label, multi-center study of improvement the outcome of patients with Heart Failure using a mobile hEalth-supported platForm

Journal:	*BMJ Open*
Manuscript ID	bmjopen-2023-081011.R1
Article Type:	Protocol
Date Submitted by the Author:	10-Jan-2024
Complete List of Authors:	Chen, Fuzhong; The First Affiliated Hospital of Nanjing Medical University, Department of Cardiology<br>Li, Guangjuan; The First Affiliated Hospital of Nanjing Medical University, Department of Cardiology; The Friendship Hospital of Ili Kazak Autonomous Prefecture, Department of Cardiology<br>Zhang, Xinxin; Northern Jiangsu People's Hospital, Department of Cardiology<br>Shen, Qin; The First Affiliated Hospital of Nanjing Medical University, Department of Cardiology<br>Wang, Fangfang; Changzhou No.2 People's Hospital, Department of Cardiology<br>Dong, Xiaoyu; The First Affiliated Hospital of Nanjing Medical University, Department of Cardiology<br>Zou, Yu; The First Affiliated Hospital of Nanjing Medical University, Department of Cardiology<br>Chen, Wensen; The First Affiliated Hospital of Nanjing Medical University, Office of Infection Management<br>Xu, Bing; Northern Jiangsu People's Hospital, Department of Cardiology<br>Wang, Junhong; The First Affiliated Hospital of Nanjing Medical University, Department of Cardiology; Jiangsu health administration and development research center
**Primary Subject Heading**:	Cardiovascular medicine
Secondary Subject Heading:	Cardiovascular medicine
Keywords:	Prognosis, Clinical Trial, Heart failure < CARDIOLOGY

SCHOLARONE™
Manuscripts

The impHFrEF trial: study protocol for an open-label, multi-center study of improvement the outcome of patients with Heart Failure using a mobile hEalth-supported platForm

Author: Chen Fuzhong[1#], Li Guangjuan[1,2#], Zhang Xinxin[3#], Shen Qin[1], Wang Fangfang[4], Dong Xiaoyu[1], Zou Yu[1], Chen Wensen[5*], Xu Bing[3]*, Wang Junhong[1,6*]

[1]Department of Cardiology, The First Affiliated Hospital of Nanjing Medical University, Nanjing, China.

[2]Department of Cardiology, The Friendship Hospital of Ili Kazak Autonomous Prefecture, Yining, China.

[3]Department of Cardiology, Northern Jiangsu People's Hospital, Yangzhou, China.

[4]Department of Cardiology, Changzhou No.2 People's Hospital, Changzhou, China.

[5]Office of Infection Management, The First Affiliated Hospital of Nanjing Medical University, Nanjing, China.

[6]Jiangsu health administration and development research center, Nanjing, China.

#These authors contributed equally.

* Corresponding to Dr. Chen Wensen: wensenchen@njmu.edu.cn, Dr. Xu Bing, Email: nyxb1980@163.com and Prof. Wang Junhong, E-mail: wangjunhong@jsph.org.cn

For peer review only

Abstract

Background

Patients with chronic heart failure (CHF) often have a long duration of illness, difficulty in attending follow-up visits, and poor adherence to treatment. As a result, they frequently cannot receive guideline-directed medical therapy (GDMT) at the desired or maximum tolerable drug dosage. This leads to high hospitalization and mortality rates for heart failure (HF) patients. Therefore, effective management and monitoring of patients with heart failure to ensure they receive GDMT is crucial for improving their prognosis.

Design and methods

This is a multicenter, open-label, randomized, parallel-group study involving patients with CHF across five centers. The study aims to assess the impact of an optimized guideline-directed medical therapy (GDMT) model for heart failure patients, established on a mobile health (mHealth) platform, compared to a control group. The primary composite outcome is worsening heart failure events (hospitalization or emergency treatment with intravenous fluids) or cardiovascular death. Patients must have a left ventricular ejection fraction (LVEF) of less than 50% and be receiving medication titration therapy that has not yet reached the target dose, with a modest increase in N-terminal pro-B-type natriuretic peptide level. The primary endpoint is a composite of cardiogenic death and hospitalization for worsening heart failure

For peer review only

symptoms.

Conclusions

The study aims to establish an optimal management model for patients with heart failure based on a mobile health (mHealth) platform. The expectation is that this model will provide an improved prognosis for patients with chronic heart failure and save social healthcare resources.

Ethics and dissemination

On December 22, 2021, this study received ethical approval from the Ethics Review Board of the First Affiliated Hospital of Nanjing Medical University, with the ethics number 2021-SR-530. All study participants will be informed of the research purpose and their participation will be voluntary. Informed consent will be obtained by providing and signing an informed consent form. We will ensure compliance with relevant laws and regulations regarding privacy and data protection. The results of this study will be published in a peer-reviewed academic journal. We will ensure that the dissemination of study results is accurate, clear, and timely.

Keywords

Clinical trial • Heart failure • Optimized management model • moblile health(mHealth) platform

For peer review only

Strengths and limitations

Strengths: (1) The study followed ethical principles and legal regulations, ensuring the safety and legitimacy of the research. (2) As a randomized controlled prospective clinical trial with a large sample size, the study provided reliable results. Limitations: (1) As a prospective clinical study, it is not possible to fully control all variables, which may have an impact on the accuracy of the research results. (2) The generalizability and applicability of the research results may be limited due to variations in medical standards across countries and regions.

Introduction

The term chronic heart failure (CHF) refers to a complex clinical syndrome resulting from abnormal changes in the structure and/or function of the heart owning to numerous factors, leading to impaired ventricular systolic and/or diastolic function [1,2]. The main manifestations are dyspnea, fatigue, and fluid retention (pulmonary circulation congestion, systemic circulation congestion, and peripheral edema) [1,3]. Most patients with acute heart failure (AHF) have partial remission of symptoms after hospitalization and are referred to chronic heart failure; Patients with chronic heart failure often require hospitalization due to acute exacerbation of various triggers. Heart failure has become an important public health issue worldwide due to its high incidence, rehospitalization rate, and death rate [4,5]. China-HF study shows a 4.1% mortality rate for hospitalized patients with heart failure in China [5]. The 2012-2015 Chinese Epidemiological

For peer review only

Survey showed that the prevalence of heart failure among adults over 35 years old in China was 1.3% (estimated 13.7 million) [6]. As China's population ages and the incidence of chronic diseases such as coronary heart disease, hypertension, and diabetes increases, improvements in medical care have led to longer survival periods for patients with heart disease, resulting in a continuing trend of increased heart failure prevalence in China.

Guideline-directed medical therapy (GDMT) is a cornerstone of care for patients with CHF. In terms of drug therapy, angiotensin-converting enzyme inhibitors (ACEIs) or angiotensin II receptor antagonists (ARBs), beta-blockers (BBs), and mineralocorticoid receptor antagonists (MRAs) constitute the standard therapy for the treatment of chronic heart failure. This can significantly improve the clinical symptoms of patients with chronic heart failure and reduce the rate of rehospitalization and mortality. Over the past few years, a variety of new heart failure therapeutic agents have emerged that can improve prognosis. Among them, extensive evidence of the clinical benefit of angiotensin receptor-neprilysin inhibitors (ARNIs) and sodium-glucose co-transporter-2 inhibitors (SGLT2is) has led to a shift from the "ACEI/ARB+BB+MRA" regimen to the " ARNI/ACEI/ARB+SGLT2i+BB+MRA " regimen for improving the prognosis of patients with heart failure with reduced ejection fraction [3,7-9].

Clinical guidelines emphasize the importance of achieving the target dose of guideline-guided medication therapy (GDMT) as much as possible [3,7]. However, due to the

For peer review only

long duration of heart failure, it is difficult for patients to be rechecked and examined after discharge due to medical and economic constraints. In addition, severe side effects such as sinus bradycardia, atrioventricular block, and blood pressure reduction can occur during β-blockers, ACEIs/ARBs, and ARNIs therapy, leading to exacerbation and recurrence of heart failure. As a result, patients' adherence to treatment is poor after discharge from the hospital, making it impossible for patients to receive the target dose of medication. This ultimately leads to high rates of rehospitalization and death from heart failure. Recent studies have shown that even in the medically advanced United States, the vast majority of patients with heart failure are not titrated to the target dose or the maximum tolerated dose of BBs and ACEIs/ARBs [10-12]. Therefore, effective follow-up and management of patients are key to improving their prognosis of patients with heart failure and saving healthcare resources.

Recent studies in China and abroad have attempted to use existing social media tools such as WeChat, Facebook, and Twitter to manage patients with coronary heart disease (CHD) and other cardiovascular diseases to effectively reduce the incidence of cardiovascular events in patients with coronary heart disease. Ge and his team reported in Lancet on the successful use of a WeChat social platform to guide the treatment of patients with CHD through refined management for rehabilitation and secondary prevention [13]. As a result, an APP has been developed for patients with chronic heart failure based on the mobile health(mHealth) platform. The APP can be used to monitor the daily condition of the patient, allowing the physician and patient to communicate in

For peer review only

real-time through the software. This establishes a remote monitoring platform with the participation of doctors and patients. By monitoring the arrhythmias of patients with heart failure and their responsiveness to medication, patients can be effectively monitored remotely and guided in the use of medication. Ultimately, patients with heart failure will be effectively managed in a timely and scientific manner, reducing the number of repeated hospitalizations, lowering the death rate of HF patients, and reducing the medical burden on society.

Consequently, the implementation of this study can establish an optimal guideline-directed medical therapy (GDMT) management model for patients with heart failure based on the mHealth platform. This model can be effectively promoted and popularized, ultimately improving the prognosis of patients with HF and saving social medical resources.

Methods

The study is a multicenter, parallel-group, randomized, open-label, prospective trial in patients with chronic heart failure. The study is scheduled to commence in June 2022 and is expected to conclude in December 2024. The registration number of the study is ChiCTR2200056527.

For peer review only

Study design and conduct

Trial Design Overview

The study is a multicenter, prospective, open-label study. A total of 5 centers participated in this study: Jiangsu Provincial People's Hospital, Northern Jiangsu People's Hospital, the Second Affiliated Hospital of Nanjing Medical University, Changzhou No.2 People's Hospital, and the Friendship Hospital of Ili Kazak Autonomous Prefecture. In each center, a team of approximately 2-3 clinical physicians is engaged, with the same doctors overseeing both trial and non-trial patients. In addition, a seasoned cardiologist conducts monthly quality control assessments at each center to ensure the rationality of drug treatment adjustments and the integrity of follow-up visits, thus ensuring the homogeneity of the study. A total of 680 patients with CHF are planned to be randomly enrolled and assigned 1:1 to the mhealth optimized GDMT group (mGDMT group) and the control group, with 340 patients in each group. The population included is heart failure medication not titrated to the target doses or maximum tolerated doses. Patients in the mGDMT group are managed individually and precisely through a mHealth platform application (app), and the dosage of beta-blockers and other HF treatment medications are titrated and adjusted to the patient's tolerated target dose according to the results of the remote mHealth platform. The control group receives the current clinical routine follow-up and treatment. In the control group, patients will be treated by experienced cardiovascular internists at the hospital, who will adjust their treatment plans according to the Chinese

For peer review only

guidelines for heart failure. The frequency of clinic visits for these patients will initially be set at 2-4 weeks, but could be extended to once every 1-3 months for those who have reached the target medication dose and have stabilized their condition. Follow-up will be performed at the intervals of 1 month, 3 months, 6 months, and 12 months after enrollment for a total of 12 months to compare clinical symptoms, cardiac function classification, incidence of adverse events, hospitalization rate, mean number of days in hospital, and mortality between the two groups. Figure 1 illustrates the trial procedures of the study.

The mHealth platform App construction

The mHealth platform App builds an interactive platform for doctors and patients to participate together. The App includes a doctor-side and a patient-side portal. The patient side of the software requires the patient to bind to the appropriate doctor and clock in daily on their weight, urine output, blood pressure, heart rates, heart failure symptoms, and other indicators (laboratory results or examination for local review). The patient-side data is uploaded and presented in a visualized way on the doctor-side. The physician understands the patients' medication reaction based on the data uploaded daily by the patient and communicates effectively with the patient through the App platform dialogue tool. The corresponding physician makes precise and standardized adjustments to the heart failure medication and treatment according to the patients' condition (by utilizing electronic prescriptions). At the same time, the platform sets up an alarm function for extreme values to alert physicians when abnormal values of key

For peer review only

indicators appear in patients, facilitating timely adjustment of patients' medication regimens. Throughout the visit, the importance of patient adherence with program requirements regarding daily measurements and symptom reporting is emphasized to ensure timely identification of potential changes in their condition. Figure 2 demonstrates the model for optimal management of patients with heart failure based on a mHealth App.

Patients

The population included in this study is patients with heart failure who have new or ongoing heart failure medication that has not yet reached target doses and requires adjustment of medication to target or goal measures, or patients with heart failure who have been treated to stop the intravenous vasoactive medication and then switched to oral medication after stabilization. The patient's left ventricular ejection fraction (LVEF) as measured by the Simpson method needs to be <50% and the New York Heart Association (NYHA) Classes II-IV (NYHA Classes IV in stable phase). The patient's brain natriuretic peptide (BNP) concentration should be ≥150 pg/ml or N-terminal pro-B-type natriuretic peptide (NT-proBNP) ≥600 pg/ml, or BNP ≥100 pg/ml if the patient has been hospitalized for heart failure within 12 months (NT-proBNP ≥400 pg/ml). Subjects must meet the age limit of 18 years, be proficient in the use of electronic devices (Proficiency in using WeChat app). Figure 3 displays the inclusion criteria for the trial.

The key exclusion criteria for the impHFrEF trial include Somatic Activity Disorders

For peer review only

and Psychiatric Disorders, Chronic obstructive pulmonary disease (COPD) and Asthma, Malignant tumor patients with a life expectancy of less than 1 year, Plans to receive device or surgical treatment such as a heart transplant, left ventricular assist device, CRTD implantation or valve replacement surgery during the follow-up period, Recurrent episodes of intractable heart failure, Various correctable secondary causes of heart failure including hyperthyroidism, anemic heart disease, congenital heart disease, organic valvular heart disease. Patients with renal failure (creatinine clearance <30 ml/min) or on dialysis treatment, hyperkalemia (blood potassium >5.5 mmol/l) at the time of enrollment and those who are participating in other clinical trials are also excluded. Figure 4 presents the exclusion criteria for the trial.

Upon providing their informed consent, eligible patients who consent to participate in this clinical trial will be randomly assigned to their respective groups. All patient data shall be held in strict confidence and accessible exclusively by authorized personnel. The usage of patients' personal information will be strictly limited to research purposes only. Under no circumstances shall patient information be utilized for any other purposes without explicit written consent from the individuals involved. At the conclusion of the investigation, all patient data will undergo meticulous anonymization procedures, guaranteeing the utmost protection of patient confidentiality.

Randomization

Once patients meet the criteria for randomization, they are assigned by investigators

For peer review only

through a central randomization system. Patients in the study are centrally randomized and competitively enrolled in either the mGDMT group or the normal control groups.

Study visits and follow-up

Patients who meet the inclusion criteria will be evaluated upon admission and randomly assigned to either the mGDMT group or the control group after signing an informed consent form. Baseline information, including clinical examination and laboratory measurements, will be collected. Follow-up visits will be conducted on days 30 ± 3, 90 ± 3, 180 ± 3, and 360 ± 3 after randomization. These visits will focus on heart failure symptoms, signs, volume status, and adverse events. ECG, echocardiography, and laboratory results such as troponin, pro-BNP, liver and kidney function will also be reviewed. The schedule for data acquisition is shown in Table 1.

Table 1 Schedule for data acquisition indicated by checkmarks at the specified time point.

Data collected	Baseline	Day 30	Day 90	Day 180	Day 360
Demographics	✓	N/A[a]	N/A	N/A	N/A
History	✓	N/A	N/A	N/A	N/A
Vital signs	✓	✓	✓	✓	✓
Signs and symptoms	✓	✓	✓	✓	✓
NYHA[b] class	✓	✓	✓	✓	✓
6-min walk test	✓	✓	✓	✓	✓
KCCQ[c] scores	✓	✓	✓	✓	✓
Laboratory results					
BNP[d] levels	✓	N/A	N/A	✓	✓

For peer review only

Troponin	✓	N/A	N/A	✓	✓
Renal function	✓	N/A	N/A	✓	✓
Liver function	✓	N/A	N/A	✓	✓
Examination results					
ECG[e]	✓	N/A	N/A	✓	✓
Echocardiography	✓	N/A	N/A	✓	✓
Endpoints					
Cardiac death	N/A	✓	✓	✓	✓
Number of HF[f]-related hospitalizations since enrollment	N/A	✓	✓	✓	✓
Number of days in the hospital since enrollment	N/A	✓	✓	✓	✓
All-cause death	N/A	✓	✓	✓	✓

[a]N/A: not applicable.

[b]NYHA: New York Heart Association.

[c]KCCQ: Kansas City Cardiomyopathy Questionnaire.

[d]BNP: brain natriuretic peptide.

[e]ECG: Electrocardiogram.

[f]HF: heart failure.

Outcomes

The primary endpoint is a composite endpoint consisting of cardiac death (deaths due to acute myocardial infarction (AMI), HF, systemic embolism, and severe arrhythmic events) and hospitalization due to worsening heart failure symptoms. Worsening heart failure symptoms are defined as the presence of heart failure that cannot be controlled

For peer review only

by oral medications and requires hospitalization or emergency room treatment with intravenous medications.

The secondary endpoints primarily include：(i) all-cause death; (ii) cumulative heart failure admissions during the trial; (iii) changes in the 6-minute walk test and heart failure biomarkers; (iv) changes from baseline to 12 months in the total symptom score using the Kansas City Cardiomyopathy Questionnaire (KCCQ) [14].

The study is expected to collect as much clinical information as possible from patients during each visit. This information will be used to compare clinical symptoms, cardiac function class, incidence of adverse events, hospitalization rate, mean days in the hospital, and mortality between the two groups of patients. The efficacy of heart failure medication and adverse events will be monitored and recorded.

Statistical considerations

Sample size assumptions

The incidence rate of endpoint events in the control group was determined based on previous research findings. The annual event rate of heart failure recurrence, cardiogenic and all-cause death in the control group is 15% [15]. The mGDMT group expected an annual event rate of 8% [16]. With a test efficacy of 0.8 and a bilateral alpha of 0.05, a sample size ratio of 1:1 for inclusion of the control and mGDMT groups, and a lost-to-review rate of 5%, a minimum sample size of 340 cases is required for

For peer review only

each of the two groups. Therefore, the study expects to include a total of 680 patients.

Methods for statistical analyses

In our experiment, the statistical methods have been meticulously preplanned. All analyses will be conducted using R (version 4.2.2) and SPSS (version 27), both of which are robust and widely accepted statistical software packages. The primary and secondary efficacy analyses will be performed on the full analysis set, adhering to the principle of intention-to-treat. This principle ensures that the results are not biased due to post-randomization exclusions. The full analysis set will include all randomized patients who have provided informed consent and have commenced the indicated treatment.

For data conforming to a normal distribution, it will be expressed as mean ± standard deviation. An independent samples t-test, a parametric test, will be employed for comparison. For variables with a skewed distribution, they will be expressed as the median and interquartile range (IQR), and a nonparametric test will be used for comparison. This approach allows us to handle data that do not meet the assumptions of parametric tests. Categorical data will be presented as counts and percentages. Chi-square or Fisher exact tests will be utilized for evaluation, depending on the distribution and characteristics of the data. The Kaplan-Meier (K-M) method, a non-parametric statistic used to estimate the survival function from lifetime data, will be used to assess the difference in the incidence of endpoint events between the two groups of patients. In addition, we will conduct a sensitivity analysis to assess the robustness of our

For peer review only

findings. A two-sided p-value less than 0.05 will be considered statistically significant. All statistical tests will be two-sided, reflecting our non-directional hypotheses.

Our statistical team, with their extensive experience and expertise, will ensure the accuracy and reliability of our analyses. By adhering to these rigorous statistical methods, we aim to produce precise and trustworthy experimental results.

Ancillary studies

After obtaining informed consent from patients, blood samples will be collected at baseline and during follow-up for the measurement of biomarkers of interest.

Trial design and governance

The study was designed by the Cardiovascular Medicine Department of the First Affiliated Hospital of Nanjing Medical University and reviewed by its Ethics Committee. The study has completed clinical trial registration and obtained a registration number. There are 5 study centers, and the study will be supervised by the ethics committees of their respective centers during the study implementation.

Patient and Public Involvement

Patients or the public were not involved in the design, or conduct, or reporting, or dissemination plans of our research.

Safety considerations

Patients with heart failure often have a changing condition, with mortality and

For peer review only

rehospitalization rates as high as 15% and 30% within 2-3 months of their discharge from the hospital [17]. Therefore, investigators need to evaluate patients' uploaded heart rates, blood pressure, heart failure symptoms, and test results to adjust therapeutic drug doses according to their hemodynamic status and fluid retention. If patients develop renal insufficiency or hyperkalemia during treatment, the medication should also be adjusted immediately according to the guidelines [3]. The trial should be terminated if there is a rapid deterioration in renal function (50% increase in creatinine level or 0.3 mg/dl increase in absolute creatinine level compared to 48 hours before) or severe sinus bradycardia requiring pacing therapy.

Current status

The First Affiliated Hospital of Nanjing Medical University ethically cleared the study on December 22, 2021, and clinical trial registration was completed on February 7, 2022. Ethics number is 2021-SR-530. The mHealth platform APP was constructed on June 1, 2022. The first subject was recruited and randomized to groups on June 29, 2022. A later phase will rapidly recruit subjects and complete randomization.

Supplementary Information

Ethics and dissemination

On December 22, 2021, this study received ethical approval from the Ethics Review Board of the First Affiliated Hospital of Nanjing Medical University, with the ethics number 2021-SR-530. All study participants will be informed of the research purpose

For peer review only

and their participation will be voluntary. Informed consent will be obtained by providing and signing an informed consent form. We will ensure compliance with relevant laws and regulations regarding privacy and data protection. The results of this study will be published in a peer-reviewed academic journal. We will ensure that the dissemination of study results is accurate, clear, and timely.

Funding

This work was supported by the National Natural Science Foundation of China (NSFC 82170269), Jiangsu Province Hospital (the first affiliated hospital with Nanjing Medical University) clinical capacity enhancement project (JSPH-MB-2021-15), the Foundation of Clinical Medical Research Center of Yili Autonomous Prefecture (YL2020ms09), and the open project of Jiangsu health administration and development research center (JSHD2022009).

Contributorship statement

Chen Fuzhong contributed to the randomization and grouping of patients, as well as the writing and revision of the paper. Li Guangjuan was responsible for patient recruitment and contributed to the paper revision. Zhang Xinxin, Shen Qin, and Wang Fangfang were involved in patient recruitment, data collection, and collation. Dong Xiaoyu assisted in patient randomization, grouping, and data collection. Zou Yu supported data collection and collation. Chen Wensen provided statistical expertise and guidance on trial design. Xu Bing offered guidance on trial design and supervised its implementation. Wang Junhong provided comprehensive guidance on trial design and implementation,

For peer review only

oversaw and coordinated the entire research process, and funded the study. Each author played a critical role in ensuring the accuracy and completeness of the research findings and is accountable for the final content of the paper.

References

1. Guan X, Sun Z. The Role of Intestinal Flora and Its Metabolites in Heart Failure. Infection and drug resistance 2023;16:51-64. (In eng). DOI: 10.2147/idr.S390582.

2. Kanodia J, Lo A, Baldwin RM, Colley K, Zhou K, Bourdet DL. Safety, Pharmacokinetics, and Pharmacodynamics of TD-0714, a Novel Potent Neprilysin Inhibitor in Healthy Adult and Elderly Subjects. Clinical and translational science 2020;13(6):1307-1315. (In eng). DOI: 10.1111/cts.12831.

3. McDonagh TA, Metra M, Adamo M, et al. 2021 ESC Guidelines for the diagnosis and treatment of acute and chronic heart failure. European heart journal 2021;42(36):3599-3726. (In eng). DOI: 10.1093/eurheartj/ehab368.

4. Chow SL, Maisel AS, Anand I, et al. Role of Biomarkers for the Prevention, Assessment, and Management of Heart Failure: A Scientific Statement From the American Heart Association. Circulation 2017;135(22):e1054-e1091. (In eng). DOI: 10.1161/cir.0000000000000490.

5. Zhang Y, Zhang J, Butler J, et al. Contemporary Epidemiology, Management, and Outcomes of Patients Hospitalized for Heart Failure in China: Results From the China Heart Failure (China-HF) Registry. Journal of cardiac failure 2017;23(12):868-875. (In eng). DOI: 10.1016/j.cardfail.2017.09.014.

For peer review only

6. Hao G, Wang X, Chen Z, et al. Prevalence of heart failure and left ventricular dysfunction in China: the China Hypertension Survey, 2012-2015. European journal of heart failure 2019;21(11):1329-1337. (In eng). DOI: 10.1002/ejhf.1629.

7. Heidenreich PA, Bozkurt B, Aguilar D, et al. 2022 AHA/ACC/HFSA Guideline for the Management of Heart Failure: Executive Summary: A Report of the American College of Cardiology/American Heart Association Joint Committee on Clinical Practice Guidelines. Circulation 2022;145(18):e876-e894. (In eng). DOI: 10.1161/cir.0000000000001062.

8. Maddox TM, Januzzi JL, Jr., Allen LA, et al. 2021 Update to the 2017 ACC Expert Consensus Decision Pathway for Optimization of Heart Failure Treatment: Answers to 10 Pivotal Issues About Heart Failure With Reduced Ejection Fraction: A Report of the American College of Cardiology Solution Set Oversight Committee. Journal of the American College of Cardiology 2021;77(6):772-810. (In eng). DOI: 10.1016/j.jacc.2020.11.022.

9. McMurray JJV, Packer M. How Should We Sequence the Treatments for Heart Failure and a Reduced Ejection Fraction?: A Redefinition of Evidence-Based Medicine. Circulation 2021;143(9):875-877. (In eng). DOI: 10.1161/circulationaha.120.052926.

10. Chang HY, Wang CC, Wei J, et al. Gap between guidelines and clinical practice in heart failure with reduced ejection fraction: Results from TSOC-HFrEF registry. Journal of the Chinese Medical Association : JCMA 2017;80(12):750-757. (In eng). DOI: 10.1016/j.jcma.2017.04.011.

11. Greene SJ, Fonarow GC, DeVore AD, et al. Titration of Medical Therapy for Heart

For peer review only

Failure With Reduced Ejection Fraction. Journal of the American College of Cardiology 2019;73(19):2365-2383. (In eng). DOI: 10.1016/j.jacc.2019.02.015.

12. Jungbauer CG, Maier LS, Emoto K, Zirille FM, Mirro MJ. Achieving Guideline-Directed Heart Rate ControlEarly Posthospitalization. The American journal of cardiology 2019;123(7):1096-1100. (In eng). DOI: 10.1016/j.amjcard.2018.12.032.

13. Dorje T, Zhao G, Tso K, et al. Smartphone and social media-based cardiac rehabilitation and secondary prevention in China (SMART-CR/SP): a parallel-group, single-blind, randomised controlled trial. The Lancet Digital health 2019;1(7):e363-e374. (In eng). DOI: 10.1016/s2589-7500(19)30151-7.

14. Green CP, Porter CB, Bresnahan DR, Spertus JA. Development and evaluation of the Kansas City Cardiomyopathy Questionnaire: a new health status measure for heart failure. Journal of the American College of Cardiology 2000;35(5):1245-55. (In eng). DOI: 10.1016/s0735-1097(00)00531-3.

15. McMurray JJ, Packer M, Desai AS, et al. Angiotensin-neprilysin inhibition versus enalapril in heart failure. The New England journal of medicine 2014;371(11):993-1004. (In eng). DOI: 10.1056/NEJMoa1409077.

16. Kotb A, Cameron C, Hsieh S, Wells G. Comparative effectiveness of different forms of telemedicine for individuals with heart failure (HF): a systematic review and network meta-analysis. PloS one 2015;10(2):e0118681. (In eng). DOI: 10.1371/journal.pone.0118681.

17. Greene SJ, Fonarow GC, Vaduganathan M, Khan SS, Butler J, Gheorghiade M. The vulnerable phase after hospitalization for heart failure. Nature reviews Cardiology

For peer review only

2015;12(4):220-9. (In eng). DOI: 10.1038/nrcardio.2015.14.

Figure 1 Trial design. CV, cardiovascular; HF, heart failure.

Figure 2 Optimized GDMT model for managing HF patients based on the mHealth platform. mHealth, mobile health; HF, heart failure; GDMT, guideline directed medical therapy.

Figure 3 Patient inclusion criteria.

Figure 4 Patient exclusion criteria.

For peer review only

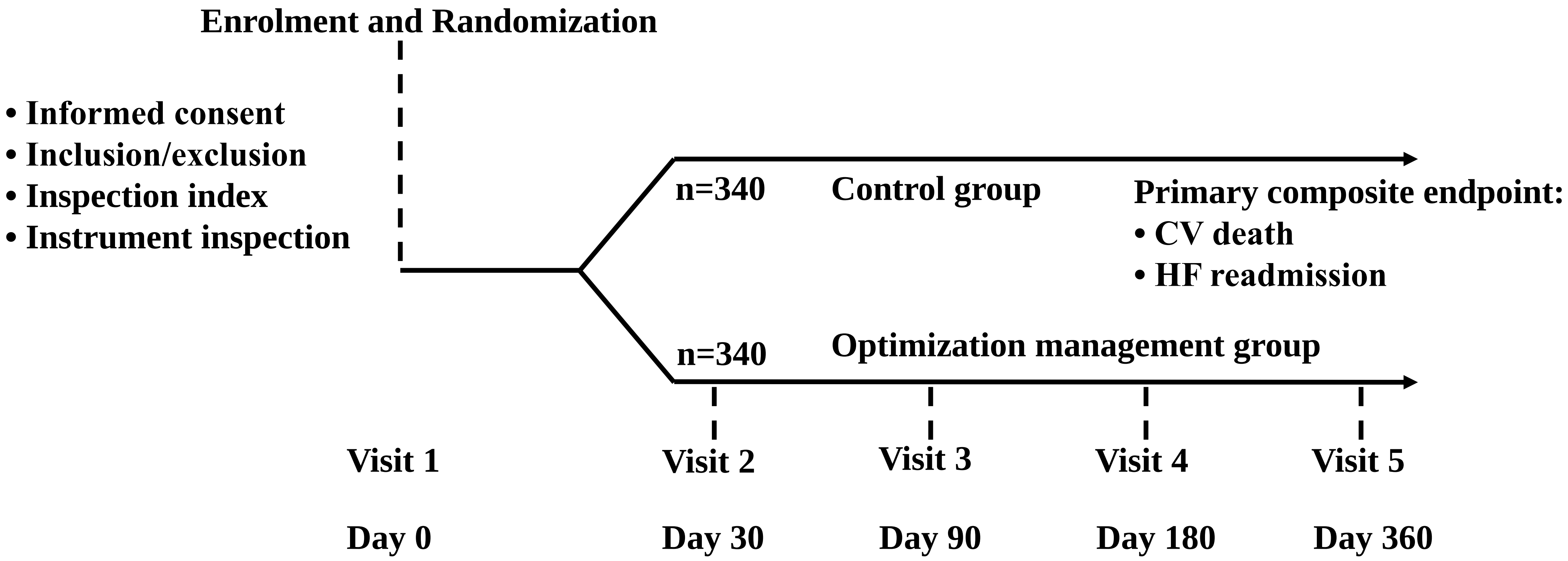
Enrolment and Randomization
• Informed consent
• Inclusion/exclusion
• Inspection index
• Instrument inspection
n=340
Control group
Primary composite endpoint:
• CV death
• HF readmission
n=340
Optimization management group
Visit 1
Visit 2
Visit 3
Visit 4
Visit 5
Day 0
Day 30
Day 90
Day 180
Day 360

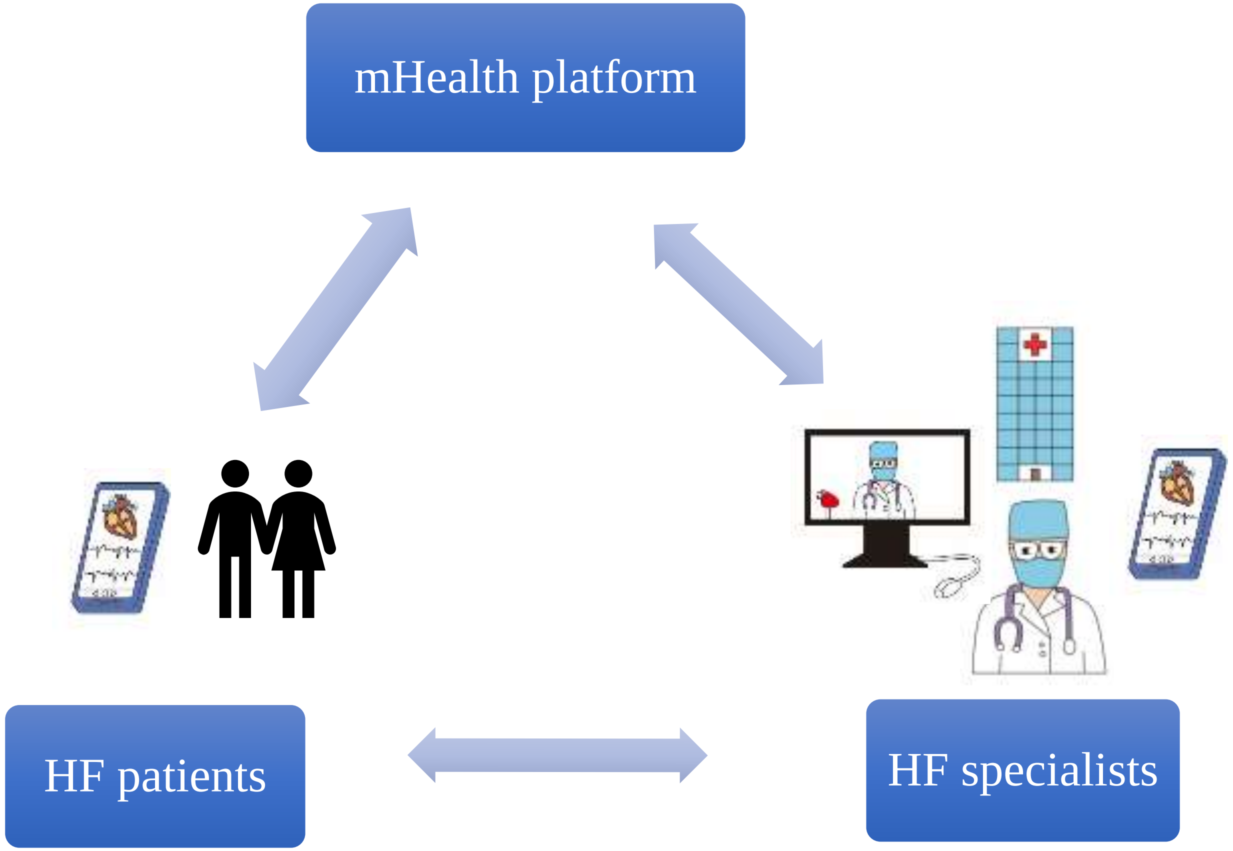
mHealth platform
HF patients
HF specialists

- Willing to provide written informed consent
- 18 years or older
- Left ventricular ejection fraction (LVEF) as measured by the Simpson method needs to be <50% and the New York Heart Association (NYHA) Classes II-IV (NYHA IV in stable phase)
- The patient's brain natriuretic peptide (BNP) concentration should be ≥150 pg/ml or N-terminal pro-B-type natriuretic peptide (NT-proBNP) ≥600 pg/ml, or BNP ≥100 pg/ml (NT-proBNP ≥400 pg/ml) if the patient has been hospitalized for heart failure within 12 months
- Heart failure patients with new or ongoing anti-heart failure medication that has not yet reached target doses and requires adjustment of medication to target or goal measures, or heart failure patients who have been treated to stop the intravenous vasoactive medication and then switched to oral medication after stabilization
- Patients should be proficient in the use of electronic devices

- Somatic Activity Disorders and Psychiatric Disorders
- Chronic obstructive pulmonary disease (COPD) and Asthma
- Malignant tumor patients (life expectancy < 1 year)
- Plans to receive device or surgical treatment (such as a heart transplant, left ventricular assist device or CRTD implantation and valve replacement surgery during the follow-up period)
- Recurrent episodes of intractable heart failure, Various correctable secondary causes of heart failure (including hyperthyroidism, anemic heart disease, congenital heart disease, organic valvular heart disease)
- Patients with renal failure (creatinine clearance <30 ml/min) or on dialysis treatment
- Hyperkalemia (blood potassium >5.5 mmol/l) at randomization
- Those who are participating in other clinical trials

Reporting checklist for protocol of a clinical trial.

Based on the SPIRIT guidelines.

Instructions to authors

Complete this checklist by entering the page numbers from your manuscript where readers will find each of the items listed below.

Your article may not currently address all the items on the checklist. Please modify your text to include the missing information. If you are certain that an item does not apply, please write "n/a" and provide a short explanation.

Upload your completed checklist as an extra file when you submit to a journal.

In your methods section, say that you used the SPIRITreporting guidelines, and cite them as:

Chan A-W, Tetzlaff JM, Gøtzsche PC, Altman DG, Mann H, Berlin J, Dickersin K, Hróbjartsson A, Schulz KF, Parulekar WR, Krleža-Jerić K, Laupacis A, Moher D. SPIRIT 2013 Explanation and Elaboration: Guidance for protocols of clinical trials. BMJ. 2013;346:e7586

		Reporting Item	Page Number
**Administrative information**			
Title	#1	Descriptive title identifying the study design, population, interventions, and, if applicable, trial acronym	1

For peer review only

Trial registration	#2a	Trial identifier and registry name. If not yet registered, name of intended registry	6
Trial registration: data set	#2b	All items from the World Health Organization Trial Registration Data Set	6
Protocol version	#3	Date and version identifier	n/a
Funding	#4	Sources and types of financial, material, and other support	16
Roles and responsibilities: contributorship	#5a	Names, affiliations, and roles of protocol contributors	1
Roles and responsibilities: sponsor contact information	#5b	Name and contact information for the trial sponsor	1,17
Roles and responsibilities: sponsor and funder	#5c	Role of study sponsor and funders, if any, in study design; collection, management, analysis, and interpretation of data; writing of the report; and the decision to submit the report for publication, including whether they will have ultimate authority over any of these activities	1,17
Roles and responsibilities: committees	#5d	Composition, roles, and responsibilities of the coordinating centre, steering committee, endpoint adjudication committee, data management team, and	13

For peer review only

		other individuals or groups overseeing the trial, if applicable (see Item 21a for data monitoring committee)	
**Introduction**			
Background and rationale	#6a	Description of research question and justification for undertaking the trial, including summary of relevant studies (published and unpublished) examining benefits and harms for each intervention	5,6
Background and rationale: choice of comparators	#6b	Explanation for choice of comparators	4-6
Objectives	#7	Specific objectives or hypotheses	6
Trial design	#8	Description of trial design including type of trial (eg, parallel group, crossover, factorial, single group), allocation ratio, and framework (eg, superiority, equivalence, non-inferiority, exploratory)	7
**Methods: Participants, interventions, and outcomes**			
Study setting	#9	Description of study settings (eg, community clinic, academic hospital) and list of countries where data will be collected. Reference to where list of study sites can be obtained	7

For peer review only

Eligibility criteria	#10	Inclusion and exclusion criteria for participants. If applicable, eligibility criteria for study centres and individuals who will perform the interventions (eg, surgeons, psychotherapists)	8、9
Interventions: description	#11a	Interventions for each group with sufficient detail to allow replication, including how and when they will be administered	7、8
Interventions: modifications	#11b	Criteria for discontinuing or modifying allocated interventions for a given trial participant (eg, drug dose change in response to harms, participant request, or improving / worsening disease)	15
Interventions: adherance	#11c	Strategies to improve adherence to intervention protocols, and any procedures for monitoring adherence (eg, drug tablet return; laboratory tests)	n/a
Interventions: concomitant care	#11d	Relevant concomitant care and interventions that are permitted or prohibited during the trial	n/a
Outcomes	#12	Primary, secondary, and other outcomes, including the specific measurement variable (eg, systolic blood pressure), analysis metric (eg, change from baseline, final value, time to event), method of aggregation (eg, median, proportion), and time point for each outcome. Explanation of the clinical relevance of chosen efficacy and harm outcomes is strongly recommended	10、11

For peer review only

Participant timeline	#13	Time schedule of enrolment, interventions (including any run-ins and washouts), assessments, and visits for participants. A schematic diagram is highly recommended (see Figure)	10
Sample size	#14	Estimated number of participants needed to achieve study objectives and how it was determined, including clinical and statistical assumptions supporting any sample size calculations	11、12
Recruitment	#15	Strategies for achieving adequate participant enrolment to reach target sample size	15,16
**Methods: Assignment of interventions (for controlled trials)**			
Allocation: sequence generation	#16a	Method of generating the allocation sequence (eg, computer-generated random numbers), and list of any factors for stratification. To reduce predictability of a random sequence, details of any planned restriction (eg, blocking) should be provided in a separate document that is unavailable to those who enrol participants or assign interventions	10
Allocation concealment mechanism	#16b	Mechanism of implementing the allocation sequence (eg, central telephone; sequentially numbered, opaque, sealed envelopes), describing any steps to conceal the sequence until interventions are assigned	10

For peer review only

Allocation: implementation	#16c	Who will generate the allocation sequence, who will enrol participants, and who will assign participants to interventions	10
Blinding (masking)	#17a	Who will be blinded after assignment to interventions (eg, trial participants, care providers, outcome assessors, data analysts), and how	6
Blinding (masking): emergency unblinding	#17b	If blinded, circumstances under which unblinding is permissible, and procedure for revealing a participant's allocated intervention during the trial	n/a
**Methods: Data collection, management, and analysis**			
Data collection plan	#18a	Plans for assessment and collection of outcome, baseline, and other trial data, including any related processes to promote data quality (eg, duplicate measurements, training of assessors) and a description of study instruments (eg, questionnaires, laboratory tests) along with their reliability and validity, if known. Reference to where data collection forms can be found, if not in the protocol	10、11
Data collection plan: retention	#18b	Plans to promote participant retention and complete follow-up, including list of any outcome data to be	10、11

For peer review only

		collected for participants who discontinue or deviate from intervention protocols	
Data management	#19	Plans for data entry, coding, security, and storage, including any related processes to promote data quality (eg, double data entry; range checks for data values). Reference to where details of data management procedures can be found, if not in the protocol	10、11
Statistics: outcomes	#20a	Statistical methods for analysing primary and secondary outcomes. Reference to where other details of the statistical analysis plan can be found, if not in the protocol	11、12
Statistics: additional analyses	#20b	Methods for any additional analyses (eg, subgroup and adjusted analyses)	11、12
Statistics: analysis population and missing data	#20c	Definition of analysis population relating to protocol non-adherence (eg, as randomised analysis), and any statistical methods to handle missing data (eg, multiple imputation)	12,13

Methods: Monitoring

Data monitoring: formal committee	#21a	Composition of data monitoring committee (DMC); summary of its role and reporting structure; statement of whether it is independent from the sponsor and competing interests; and reference to where further details about its charter can be found, if not in the	13

For peer review only

		protocol. Alternatively, an explanation of why a DMC is not needed	
Data monitoring: interim analysis	#21b	Description of any interim analyses and stopping guidelines, including who will have access to these interim results and make the final decision to terminate the trial	13
Harms	#22	Plans for collecting, assessing, reporting, and managing solicited and spontaneously reported adverse events and other unintended effects of trial interventions or trial conduct	15
Auditing	#23	Frequency and procedures for auditing trial conduct, if any, and whether the process will be independent from investigators and the sponsor	n/a
**Ethics and dissemination**			
Research ethics approval	#24	Plans for seeking research ethics committee / institutional review board (REC / IRB) approval	15,16
Protocol amendments	#25	Plans for communicating important protocol modifications (eg, changes to eligibility criteria, outcomes, analyses) to relevant parties (eg, investigators, REC / IRBs, trial participants, trial registries, journals, regulators)	n/a

For peer review only

Consent or assent	#26a	Who will obtain informed consent or assent from potential trial participants or authorised surrogates, and how (see Item 32)	9
Consent or assent: ancillary studies	#26b	Additional consent provisions for collection and use of participant data and biological specimens in ancillary studies, if applicable	12
Confidentiality	#27	How personal information about potential and enrolled participants will be collected, shared, and maintained in order to protect confidentiality before, during, and after the trial	10
Declaration of interests	#28	Financial and other competing interests for principal investigators for the overall trial and each study site	17
Data access	#29	Statement of who will have access to the final trial dataset, and disclosure of contractual agreements that limit such access for investigators	n/a
Ancillary and post trial care	#30	Provisions, if any, for ancillary and post-trial care, and for compensation to those who suffer harm from trial participation	15
Dissemination policy: trial results	#31a	Plans for investigators and sponsor to communicate trial results to participants, healthcare professionals, the public, and other relevant groups (eg, via publication, reporting in results databases, or other data sharing arrangements), including any publication restrictions	n/a

For peer review only

Dissemination policy: authorship	#31b	Authorship eligibility guidelines and any intended use of professional writers	1
Dissemination policy: reproducible research	#31c	Plans, if any, for granting public access to the full protocol, participant-level dataset, and statistical code	n/a

Appendices

Informed consent materials	#32	Model consent form and other related documentation given to participants and authorised surrogates	12、13
Biological specimens	#33	Plans for collection, laboratory evaluation, and storage of biological specimens for genetic or molecular analysis in the current trial and for future use in ancillary studies, if applicable	12、13

None The SPIRIT Explanation and Elaboration paper is distributed under the terms of the Creative Commons Attribution License CC-BY-NC. This checklist can be completed online using https://www.goodreports.org/, a tool made by the EQUATOR Network in collaboration with Penelope.ai

For peer review only

BMJ Open

The impHFrEF trial: study protocol for an open-label, multi-center study of improvement the outcome of patients with Heart Failure in China using a mobile hEalth-supported platForm

Journal:	*BMJ Open*
Manuscript ID	bmjopen-2023-081011.R2
Article Type:	Protocol
Date Submitted by the Author:	31-Jan-2024
Complete List of Authors:	Chen, Fuzhong; The First Affiliated Hospital of Nanjing Medical University, Department of Cardiology<br>Li, Guangjuan; The First Affiliated Hospital of Nanjing Medical University, Department of Cardiology; The Friendship Hospital of Ili Kazak Autonomous Prefecture, Department of Cardiology<br>Zhang, Xinxin; Northern Jiangsu People's Hospital, Department of Cardiology<br>Shen, Qin; The First Affiliated Hospital of Nanjing Medical University, Department of Cardiology<br>Wang, Fangfang; Changzhou No.2 People's Hospital, Department of Cardiology<br>Dong, Xiaoyu; The First Affiliated Hospital of Nanjing Medical University, Department of Cardiology<br>Zou, Yu; The First Affiliated Hospital of Nanjing Medical University, Department of Cardiology<br>Chen, Wensen; The First Affiliated Hospital of Nanjing Medical University, Office of Infection Management<br>Xu, Bing; Northern Jiangsu People's Hospital, Department of Cardiology<br>Wang, Junhong; The First Affiliated Hospital With Nanjing Medical University, Department of Cardiology; Jiangsu health administration and development research center
**Primary Subject Heading**:	Cardiovascular medicine
Secondary Subject Heading:	Cardiovascular medicine
Keywords:	Prognosis, Clinical Trial, Heart failure < CARDIOLOGY

SCHOLARONE™
Manuscripts

The impHFrEF trial: study protocol for an open-label, multi-center study of improvement the outcome of patients with Heart Failure in China using a mobile hEalth-supported platForm

Author: Chen Fuzhong[1#], Li Guangjuan[1,2#], Zhang Xinxin[3#], Shen Qin[1], Wang Fangfang[4], Dong Xiaoyu[1], Zou Yu[1], Chen Wensen[5*], Xu Bing[3]*, Wang Junhong[1,6*]

[1]Department of Cardiology, The First Affiliated Hospital of Nanjing Medical University, Nanjing, China.

[2]Department of Cardiology, The Friendship Hospital of Ili Kazak Autonomous Prefecture, Yining, China.

[3]Department of Cardiology, Northern Jiangsu People's Hospital, Yangzhou, China.

[4]Department of Cardiology, Changzhou No.2 People's Hospital, Changzhou, China.

[5]Office of Infection Management, The First Affiliated Hospital of Nanjing Medical University, Nanjing, China.

[6]Jiangsu health administration and development research center, Nanjing, China.

#These authors contributed equally.

* Corresponding to Dr. Chen Wensen: wensenchen@njmu.edu.cn, Dr. Xu Bing, Email: nyxb1980@163.com and Prof. Wang Junhong, E-mail: wangjunhong@jsph.org.cn

For peer review only

Abstract

Background

Patients with chronic heart failure (CHF) often have a long duration of illness, difficulty in attending follow-up visits, and poor adherence to treatment. As a result, they frequently cannot receive guideline-directed medical therapy (GDMT) at the desired or maximum tolerable drug dosage. This leads to high hospitalization and mortality rates for heart failure (HF) patients. Therefore, effective management and monitoring of patients with heart failure to ensure they receive GDMT is crucial for improving their prognosis.

Design and methods

This is a multicenter, open-label, randomized, parallel-group study involving patients with CHF across five centers. The study aims to assess the impact of an optimized guideline-directed medical therapy (GDMT) model for heart failure patients, established on a mobile health (mHealth) platform, compared to a control group. The primary composite outcome is worsening heart failure events (hospitalization or emergency treatment with intravenous fluids) or cardiovascular death. Patients must have a left ventricular ejection fraction (LVEF) of less than 50% and be receiving medication titration therapy that has not yet reached the target dose, with a modest increase in N-terminal pro-B-type natriuretic peptide level. The primary endpoint is a composite of cardiogenic death and hospitalization for worsening heart failure

For peer review only

symptoms.

Ethics and dissemination

On December 22, 2021, this study received ethical approval from the Ethics Review Board of the First Affiliated Hospital of Nanjing Medical University, with the ethics number 2021-SR-530. All study participants will be informed of the research purpose and their participation will be voluntary. Informed consent will be obtained by providing and signing an informed consent form. We will ensure compliance with relevant laws and regulations regarding privacy and data protection. The results of this study will be published in a peer-reviewed academic journal. We will ensure that the dissemination of study results is accurate, clear, and timely.

Keywords

Clinical trial • Heart failure • Optimized management model • moblile health(mHealth) platform

Strengths and limitations

Strengths: (1) The study followed ethical principles and legal regulations, ensuring the safety and legitimacy of the research. (2) As a randomized controlled prospective clinical trial with a large sample size, the study provided reliable results. Limitations: (1) As a prospective clinical study, it is not possible to fully control all variables, which may have an impact on the accuracy of the research results. (2) The generalizability

For peer review only

and applicability of the research results may be limited due to variations in medical standards across countries and regions.

Introduction

The term chronic heart failure (CHF) refers to a complex clinical syndrome resulting from abnormal changes in the structure and/or function of the heart owning to numerous factors, leading to impaired ventricular systolic and/or diastolic function [1,2]. The main manifestations are dyspnea, fatigue, and fluid retention (pulmonary circulation congestion, systemic circulation congestion, and peripheral edema) [1,3]. Most patients with acute heart failure (AHF) have partial remission of symptoms after hospitalization and are referred to chronic heart failure; Patients with chronic heart failure often require hospitalization due to acute exacerbation of various triggers. Heart failure has become an important public health issue worldwide due to its high incidence, rehospitalization rate, and death rate [4,5]. China-HF study shows a 4.1% mortality rate for hospitalized patients with heart failure in China [5]. The 2012-2015 Chinese Epidemiological Survey showed that the prevalence of heart failure among adults over 35 years old in China was 1.3% (estimated 13.7 million) [6]. As China's population ages and the incidence of chronic diseases such as coronary heart disease, hypertension, and diabetes increases, improvements in medical care have led to longer survival periods for patients with heart disease, resulting in a continuing trend of increased heart failure prevalence in China.

For peer review only

Guideline-directed medical therapy (GDMT) is a cornerstone of care for patients with CHF. In terms of drug therapy, angiotensin-converting enzyme inhibitors (ACEIs) or angiotensin II receptor antagonists (ARBs), beta-blockers (BBs), and mineralocorticoid receptor antagonists (MRAs) constitute the standard therapy for the treatment of chronic heart failure. This can significantly improve the clinical symptoms of patients with chronic heart failure and reduce the rate of rehospitalization and mortality. Over the past few years, a variety of new heart failure therapeutic agents have emerged that can improve prognosis. Among them, extensive evidence of the clinical benefit of angiotensin receptor-neprilysin inhibitors (ARNIs) and sodium-glucose co-transporter-2 inhibitors (SGLT2is) has led to a shift from the "ACEI/ARB+BB+MRA" regimen to the " ARNI/ACEI/ARB+SGLT2i+BB+MRA " regimen for improving the prognosis of patients with heart failure with reduced ejection fraction [3,7-9].

Clinical guidelines emphasize the importance of achieving the target dose of guideline-guided medication therapy (GDMT) as much as possible [3,7]. However, due to the long duration of heart failure, it is difficult for patients to be rechecked and examined after discharge due to medical and economic constraints. In addition, severe side effects such as sinus bradycardia, atrioventricular block, and blood pressure reduction can occur during β-blockers, ACEIs/ARBs, and ARNIs therapy, leading to exacerbation and recurrence of heart failure. As a result, patients' adherence to treatment is poor after discharge from the hospital, making it impossible for patients to receive the target dose of medication. This ultimately leads to high rates of rehospitalization and death from

For peer review only

heart failure. Recent studies have shown that even in the medically advanced United States, the vast majority of patients with heart failure are not titrated to the target dose or the maximum tolerated dose of BBs and ACEIs/ARBs [10-12]. Therefore, effective follow-up and management of patients are key to improving their prognosis of patients with heart failure and saving healthcare resources.

Recent studies in China and abroad have attempted to use existing social media tools such as WeChat, Facebook, and Twitter to manage patients with coronary heart disease (CHD) and other cardiovascular diseases to effectively reduce the incidence of cardiovascular events in patients with coronary heart disease. Ge and his team reported in Lancet on the successful use of a WeChat social platform to guide the treatment of patients with CHD through refined management for rehabilitation and secondary prevention [13]. As a result, an APP has been developed for patients with chronic heart failure based on the mobile health(mHealth) platform. The APP can be used to monitor the daily condition of the patient, allowing the physician and patient to communicate in real-time through the software. This establishes a remote monitoring platform with the participation of doctors and patients. By monitoring the arrhythmias of patients with heart failure and their responsiveness to medication, patients can be effectively monitored remotely and guided in the use of medication. Ultimately, patients with heart failure will be effectively managed in a timely and scientific manner, reducing the number of repeated hospitalizations, lowering the death rate of HF patients, and reducing the medical burden on society.

For peer review only

Consequently, the implementation of this study can establish an optimal guideline-directed medical therapy (GDMT) management model for patients with heart failure based on the mHealth platform. This model can be effectively promoted and popularized, ultimately improving the prognosis of patients with HF and saving social medical resources.

Methods

The study is a multicenter, parallel-group, randomized, open-label, prospective trial in patients with chronic heart failure. The study is scheduled to commence in June 2022 and is expected to conclude in December 2024. The registration number of the study is ChiCTR2200056527.

Study design and conduct

Trial Design Overview

The study is a multicenter, prospective, open-label study. A total of 5 centers participated in this study: Jiangsu Provincial People's Hospital, Northern Jiangsu People's Hospital, the Second Affiliated Hospital of Nanjing Medical University, Changzhou No.2 People’s Hospital, and the Friendship Hospital of Ili Kazak Autonomous Prefecture. In each center, a team of approximately 2-3 clinical physicians is engaged, with the same doctors overseeing both trial and non-trial patients. In addition, a seasoned cardiologist conducts monthly quality control assessments at each

For peer review only

center to ensure the rationality of drug treatment adjustments and the integrity of follow-up visits, thus ensuring the homogeneity of the study. A total of 680 patients with CHF are planned to be randomly enrolled and assigned 1:1 to the mhealth optimized GDMT group (mGDMT group) and the control group, with 340 patients in each group. The population included is heart failure medication not titrated to the target doses or maximum tolerated doses. Patients in the mGDMT group are managed individually and precisely through a mHealth platform application (app), and the dosage of beta-blockers and other HF treatment medications are titrated and adjusted to the patient's tolerated target dose according to the results of the remote mHealth platform. The control group receives the current clinical routine follow-up and treatment. In the control group, patients will be treated by experienced cardiovascular internists at the hospital, who will adjust their treatment plans according to the Chinese guidelines for heart failure. The frequency of clinic visits for these patients will initially be set at 2-4 weeks, but could be extended to once every 1-3 months for those who have reached the target medication dose and have stabilized their condition. Follow-up will be performed at the intervals of 1 month, 3 months, 6 months, and 12 months after enrollment for a total of 12 months to compare clinical symptoms, cardiac function classification, incidence of adverse events, hospitalization rate, mean number of days in hospital, and mortality between the two groups. Figure 1 illustrates the trial procedures of the study.

For peer review only

The mHealth platform App construction

The mHealth platform App builds an interactive platform for doctors and patients to participate together. The App includes a doctor-side and a patient-side portal. The patient side of the software requires the patient to bind to the appropriate doctor and clock in daily on their weight, urine output, blood pressure, heart rates, heart failure symptoms, and other indicators (laboratory results or examination for local review). The patient-side data is uploaded and presented in a visualized way on the doctor-side. The physician understands the patients' medication reaction based on the data uploaded daily by the patient and communicates effectively with the patient through the App platform dialogue tool. The corresponding physician makes precise and standardized adjustments to the heart failure medication and treatment according to the patients' condition (by utilizing electronic prescriptions). At the same time, the platform sets up an alarm function for extreme values to alert physicians when abnormal values of key indicators appear in patients, facilitating timely adjustment of patients' medication regimens. Throughout the visit, the importance of patient adherence with program requirements regarding daily measurements and symptom reporting is emphasized to ensure timely identification of potential changes in their condition. Figure 2 demonstrates the model for optimal management of patients with heart failure based on a mHealth App.

Patients

The population included in this study is patients with heart failure who have new or

For peer review only

ongoing heart failure medication that has not yet reached target doses and requires adjustment of medication to target or goal measures, or patients with heart failure who have been treated to stop the intravenous vasoactive medication and then switched to oral medication after stabilization. The patient's left ventricular ejection fraction (LVEF) as measured by the Simpson method needs to be <50% and the New York Heart Association (NYHA) Classes II-IV (NYHA Classes IV in stable phase). The patient's brain natriuretic peptide (BNP) concentration should be ≥150 pg/ml or N-terminal pro-B-type natriuretic peptide (NT-proBNP) ≥600 pg/ml, or BNP ≥100 pg/ml if the patient has been hospitalized for heart failure within 12 months (NT-proBNP ≥400 pg/ml). Subjects must meet the age limit of 18 years, be proficient in the use of electronic devices (Proficiency in using WeChat app). Figure 3 displays the inclusion criteria for the trial.

The key exclusion criteria for the impHFrEF trial include Somatic Activity Disorders and Psychiatric Disorders, Chronic obstructive pulmonary disease (COPD) and Asthma, Malignant tumor patients with a life expectancy of less than 1 year, Plans to receive device or surgical treatment such as a heart transplant, left ventricular assist device, CRTD implantation or valve replacement surgery during the follow-up period, Recurrent episodes of intractable heart failure, Various correctable secondary causes of heart failure including hyperthyroidism, anemic heart disease, congenital heart disease, organic valvular heart disease. Patients with renal failure (creatinine clearance <30 ml/min) or on dialysis treatment, hyperkalemia (blood potassium >5.5 mmol/l) at the time of enrollment and those who are participating in other clinical trials are also

For peer review only

excluded. Figure 4 presents the exclusion criteria for the trial.

Upon providing their informed consent, eligible patients who consent to participate in this clinical trial will be randomly assigned to their respective groups. All patient data shall be held in strict confidence and accessible exclusively by authorized personnel. The usage of patients' personal information will be strictly limited to research purposes only. Under no circumstances shall patient information be utilized for any other purposes without explicit written consent from the individuals involved. At the conclusion of the investigation, all patient data will undergo meticulous anonymization procedures, guaranteeing the utmost protection of patient confidentiality.

Randomization

Once patients meet the criteria for randomization, they are assigned by investigators through a central randomization system. Patients in the study are centrally randomized and competitively enrolled in either the mGDMT group or the normal control groups.

Study visits and follow-up

Patients who meet the inclusion criteria will be evaluated upon admission and randomly assigned to either the mGDMT group or the control group after signing an informed consent form. Baseline information, including clinical examination and laboratory measurements, will be collected. Follow-up visits will be conducted on days 30 ± 3, 90 ± 3, 180 ± 3, and 360 ± 3 after randomization. These visits will focus on heart failure

For peer review only

symptoms, signs, volume status, and adverse events. ECG, echocardiography, and laboratory results such as troponin, pro-BNP, liver and kidney function will also be reviewed. The schedule for data acquisition is shown in Table 1.

Table 1 Schedule for data acquisition indicated by checkmarks at the specified time point.

Data collected	Baseline	Day 30	Day 90	Day 180	Day 360
Demographics	✓	N/A[a]	N/A	N/A	N/A
History	✓	N/A	N/A	N/A	N/A
Vital signs	✓	✓	✓	✓	✓
Signs and symptoms	✓	✓	✓	✓	✓
NYHA[b] class	✓	✓	✓	✓	✓
6-min walk test	✓	✓	✓	✓	✓
KCCQ[c] scores	✓	✓	✓	✓	✓
Laboratory results					
BNP[d] levels	✓	N/A	N/A	✓	✓
Troponin	✓	N/A	N/A	✓	✓
Renal function	✓	N/A	N/A	✓	✓
Liver function	✓	N/A	N/A	✓	✓
Examination results					
ECG[e]	✓	N/A	N/A	✓	✓
Echocardiography	✓	N/A	N/A	✓	✓
Endpoints					
Cardiac death	N/A	✓	✓	✓	✓
Number of HF[f]-related hospitalizations since enrollment	N/A	✓	✓	✓	✓

For peer review only

Number of days in the hospital since enrollment	N/A	✓	✓	✓	✓
All-cause death	N/A	✓	✓	✓	✓

[a]N/A: not applicable.

[b]NYHA: New York Heart Association.

[c]KCCQ: Kansas City Cardiomyopathy Questionnaire.

[d]BNP: brain natriuretic peptide.

[e]ECG: Electrocardiogram.

[f]HF: heart failure.

Outcomes

The primary endpoint is a composite endpoint consisting of cardiac death (deaths due to acute myocardial infarction (AMI), HF, systemic embolism, and severe arrhythmic events) and hospitalization due to worsening heart failure symptoms. Worsening heart failure symptoms are defined as the presence of heart failure that cannot be controlled by oral medications and requires hospitalization or emergency room treatment with intravenous medications.

The secondary endpoints primarily include：(i) all-cause death; (ii) cumulative heart failure admissions during the trial; (iii) changes in the 6-minute walk test and heart failure biomarkers; (iv) changes from baseline to 12 months in the total symptom score using the Kansas City Cardiomyopathy Questionnaire (KCCQ) [14].

For peer review only

The study is expected to collect as much clinical information as possible from patients during each visit. This information will be used to compare clinical symptoms, cardiac function class, incidence of adverse events, hospitalization rate, mean days in the hospital, and mortality between the two groups of patients. The efficacy of heart failure medication and adverse events will be monitored and recorded.

Statistical considerations

Sample size assumptions

The incidence rate of endpoint events in the control group was determined based on previous research findings. The annual event rate of heart failure recurrence, cardiogenic and all-cause death in the control group is 15% [15]. The mGDMT group expected an annual event rate of 8% [16]. With a test efficacy of 0.8 and a bilateral alpha of 0.05, a sample size ratio of 1:1 for inclusion of the control and mGDMT groups, and a lost-to-review rate of 5%, a minimum sample size of 340 cases is required for each of the two groups. Therefore, the study expects to include a total of 680 patients.

Methods for statistical analyses

In our experiment, the statistical methods have been meticulously preplanned. All analyses will be conducted using R (version 4.2.2) and SPSS (version 27), both of which are robust and widely accepted statistical software packages. The primary and secondary efficacy analyses will be performed on the full analysis set, adhering to the principle of intention-to-treat. This principle ensures that the results are not biased due

For peer review only

to post-randomization exclusions. The full analysis set will include all randomized patients who have provided informed consent and have commenced the indicated treatment.

For data conforming to a normal distribution, it will be expressed as mean ± standard deviation. An independent samples t-test, a parametric test, will be employed for comparison. For variables with a skewed distribution, they will be expressed as the median and interquartile range (IQR), and a nonparametric test will be used for comparison. This approach allows us to handle data that do not meet the assumptions of parametric tests. Categorical data will be presented as counts and percentages. Chi-square or Fisher exact tests will be utilized for evaluation, depending on the distribution and characteristics of the data. The Kaplan-Meier (K-M) method, a non-parametric statistic used to estimate the survival function from lifetime data, will be used to assess the difference in the incidence of endpoint events between the two groups of patients. In addition, we will conduct a sensitivity analysis to assess the robustness of our findings. A two-sided p-value less than 0.05 will be considered statistically significant. All statistical tests will be two-sided, reflecting our non-directional hypotheses.

Our statistical team, with their extensive experience and expertise, will ensure the accuracy and reliability of our analyses. By adhering to these rigorous statistical methods, we aim to produce precise and trustworthy experimental results.

Ancillary studies

After obtaining informed consent from patients, blood samples will be collected at

For peer review only

baseline and during follow-up for the measurement of biomarkers of interest.

Trial design and governance

The study was designed by the Cardiovascular Medicine Department of the First Affiliated Hospital of Nanjing Medical University and reviewed by its Ethics Committee. The study has completed clinical trial registration and obtained a registration number. There are 5 study centers, and the study will be supervised by the ethics committees of their respective centers during the study implementation.

Patient and Public Involvement

Patients or the public were not involved in the design, or conduct, or reporting, or dissemination plans of our research.

Safety considerations

Patients with heart failure often have a changing condition, with mortality and rehospitalization rates as high as 15% and 30% within 2-3 months of their discharge from the hospital [17]. Therefore, investigators need to evaluate patients' uploaded heart rates, blood pressure, heart failure symptoms, and test results to adjust therapeutic drug doses according to their hemodynamic status and fluid retention. If patients develop renal insufficiency or hyperkalemia during treatment, the medication should also be adjusted immediately according to the guidelines [3]. The trial should be terminated if there is a rapid deterioration in renal function (50% increase in creatinine level or 0.3

For peer review only

mg/dl increase in absolute creatinine level compared to 48 hours before) or severe sinus bradycardia requiring pacing therapy.

Current status

The First Affiliated Hospital of Nanjing Medical University ethically cleared the study on December 22, 2021, and clinical trial registration was completed on February 7, 2022. Ethics number is 2021-SR-530. The mHealth platform APP was constructed on June 1, 2022. The first subject was recruited and randomized to groups on June 29, 2022. A later phase will rapidly recruit subjects and complete randomization.

Supplementary Information

Ethics and dissemination

On December 22, 2021, this study received ethical approval from the Ethics Review Board of the First Affiliated Hospital of Nanjing Medical University, with the ethics number 2021-SR-530. All study participants will be informed of the research purpose and their participation will be voluntary. Informed consent will be obtained by providing and signing an informed consent form. We will ensure compliance with relevant laws and regulations regarding privacy and data protection. The results of this study will be published in a peer-reviewed academic journal. We will ensure that the dissemination of study results is accurate, clear, and timely.

For peer review only

Funding

This work was supported by the National Natural Science Foundation of China (NSFC 82170269), Jiangsu Province Hospital (the first affiliated hospital with Nanjing Medical University) clinical capacity enhancement project (JSPH-MB-2021-15), the Foundation of Clinical Medical Research Center of Yili Autonomous Prefecture (YL2020ms09), and the open project of Jiangsu health administration and development research center (JSHD2022009).

Contributorship statement

Chen Fuzhong contributed to the randomization and grouping of patients, as well as the writing and revision of the paper. Li Guangjuan was responsible for patient recruitment and contributed to the paper revision. Zhang Xinxin, Shen Qin, and Wang Fangfang were involved in patient recruitment, data collection, and collation. Dong Xiaoyu assisted in patient randomization, grouping, and data collection. Zou Yu supported data collection and collation. Chen Wensen provided statistical expertise and guidance on trial design. Xu Bing offered guidance on trial design and supervised its implementation. Wang Junhong provided comprehensive guidance on trial design and implementation, oversaw and coordinated the entire research process, and funded the study. Each author played a critical role in ensuring the accuracy and completeness of the research findings and is accountable for the final content of the paper.

For peer review only

Competing Interests Statement

All authors have contributed significantly to the creation of this manuscript and have approved the final version. We affirm that there are no competing interests, financial or otherwise, that could influence the interpretation or presentation of the results presented in this work.

References

1. Guan X, Sun Z. The Role of Intestinal Flora and Its Metabolites in Heart Failure. Infection and drug resistance 2023;16:51-64. (In eng). DOI: 10.2147/idr.S390582.
2. Kanodia J, Lo A, Baldwin RM, Colley K, Zhou K, Bourdet DL. Safety, Pharmacokinetics, and Pharmacodynamics of TD-0714, a Novel Potent Neprilysin Inhibitor in Healthy Adult and Elderly Subjects. Clinical and translational science 2020;13(6):1307-1315. (In eng). DOI: 10.1111/cts.12831.
3. McDonagh TA, Metra M, Adamo M, et al. 2021 ESC Guidelines for the diagnosis and treatment of acute and chronic heart failure. European heart journal 2021;42(36):3599-3726. (In eng). DOI: 10.1093/eurheartj/ehab368.
4. Chow SL, Maisel AS, Anand I, et al. Role of Biomarkers for the Prevention, Assessment, and Management of Heart Failure: A Scientific Statement From the American Heart Association. Circulation 2017;135(22):e1054-e1091. (In eng). DOI: 10.1161/cir.0000000000000490.
5. Zhang Y, Zhang J, Butler J, et al. Contemporary Epidemiology, Management, and Outcomes of Patients Hospitalized for Heart Failure in China: Results From the China

For peer review only

Heart Failure (China-HF) Registry. Journal of cardiac failure 2017;23(12):868-875. (In eng). DOI: 10.1016/j.cardfail.2017.09.014.

6. Hao G, Wang X, Chen Z, et al. Prevalence of heart failure and left ventricular dysfunction in China: the China Hypertension Survey, 2012-2015. European journal of heart failure 2019;21(11):1329-1337. (In eng). DOI: 10.1002/ejhf.1629.

7. Heidenreich PA, Bozkurt B, Aguilar D, et al. 2022 AHA/ACC/HFSA Guideline for the Management of Heart Failure: Executive Summary: A Report of the American College of Cardiology/American Heart Association Joint Committee on Clinical Practice Guidelines. Circulation 2022;145(18):e876-e894. (In eng). DOI: 10.1161/cir.0000000000001062.

8. Maddox TM, Januzzi JL, Jr., Allen LA, et al. 2021 Update to the 2017 ACC Expert Consensus Decision Pathway for Optimization of Heart Failure Treatment: Answers to 10 Pivotal Issues About Heart Failure With Reduced Ejection Fraction: A Report of the American College of Cardiology Solution Set Oversight Committee. Journal of the American College of Cardiology 2021;77(6):772-810. (In eng). DOI: 10.1016/j.jacc.2020.11.022.

9. McMurray JJV, Packer M. How Should We Sequence the Treatments for Heart Failure and a Reduced Ejection Fraction?: A Redefinition of Evidence-Based Medicine. Circulation 2021;143(9):875-877. (In eng). DOI: 10.1161/circulationaha.120.052926.

10. Chang HY, Wang CC, Wei J, et al. Gap between guidelines and clinical practice in heart failure with reduced ejection fraction: Results from TSOC-HFrEF registry. Journal of the Chinese Medical Association : JCMA 2017;80(12):750-757. (In eng). DOI:

For peer review only

10.1016/j.jcma.2017.04.011.

11. Greene SJ, Fonarow GC, DeVore AD, et al. Titration of Medical Therapy for Heart Failure With Reduced Ejection Fraction. Journal of the American College of Cardiology 2019;73(19):2365-2383. (In eng). DOI: 10.1016/j.jacc.2019.02.015.

12. Jungbauer CG, Maier LS, Emoto K, Zirille FM, Mirro MJ. Achieving Guideline-Directed Heart Rate ControlEarly Posthospitalization. The American journal of cardiology 2019;123(7):1096-1100. (In eng). DOI: 10.1016/j.amjcard.2018.12.032.

13. Dorje T, Zhao G, Tso K, et al. Smartphone and social media-based cardiac rehabilitation and secondary prevention in China (SMART-CR/SP): a parallel-group, single-blind, randomised controlled trial. The Lancet Digital health 2019;1(7):e363-e374. (In eng). DOI: 10.1016/s2589-7500(19)30151-7.

14. Green CP, Porter CB, Bresnahan DR, Spertus JA. Development and evaluation of the Kansas City Cardiomyopathy Questionnaire: a new health status measure for heart failure. Journal of the American College of Cardiology 2000;35(5):1245-55. (In eng). DOI: 10.1016/s0735-1097(00)00531-3.

15. McMurray JJ, Packer M, Desai AS, et al. Angiotensin-neprilysin inhibition versus enalapril in heart failure. The New England journal of medicine 2014;371(11):993-1004. (In eng). DOI: 10.1056/NEJMoa1409077.

16. Kotb A, Cameron C, Hsieh S, Wells G. Comparative effectiveness of different forms of telemedicine for individuals with heart failure (HF): a systematic review and network meta-analysis. PloS one 2015;10(2):e0118681. (In eng). DOI: 10.1371/journal.pone.0118681.

For peer review only

17. Greene SJ, Fonarow GC, Vaduganathan M, Khan SS, Butler J, Gheorghiade M. The vulnerable phase after hospitalization for heart failure. Nature reviews Cardiology 2015;12(4):220-9. (In eng). DOI: 10.1038/nrcardio.2015.14.

Figure 1 Trial design. CV, cardiovascular; HF, heart failure.

Figure 2 Optimized GDMT model for managing HF patients based on the mHealth platform. mHealth, mobile health; HF, heart failure; GDMT, guideline directed medical therapy.

Figure 3 Patient inclusion criteria.

Figure 4 Patient exclusion criteria.

For peer review only

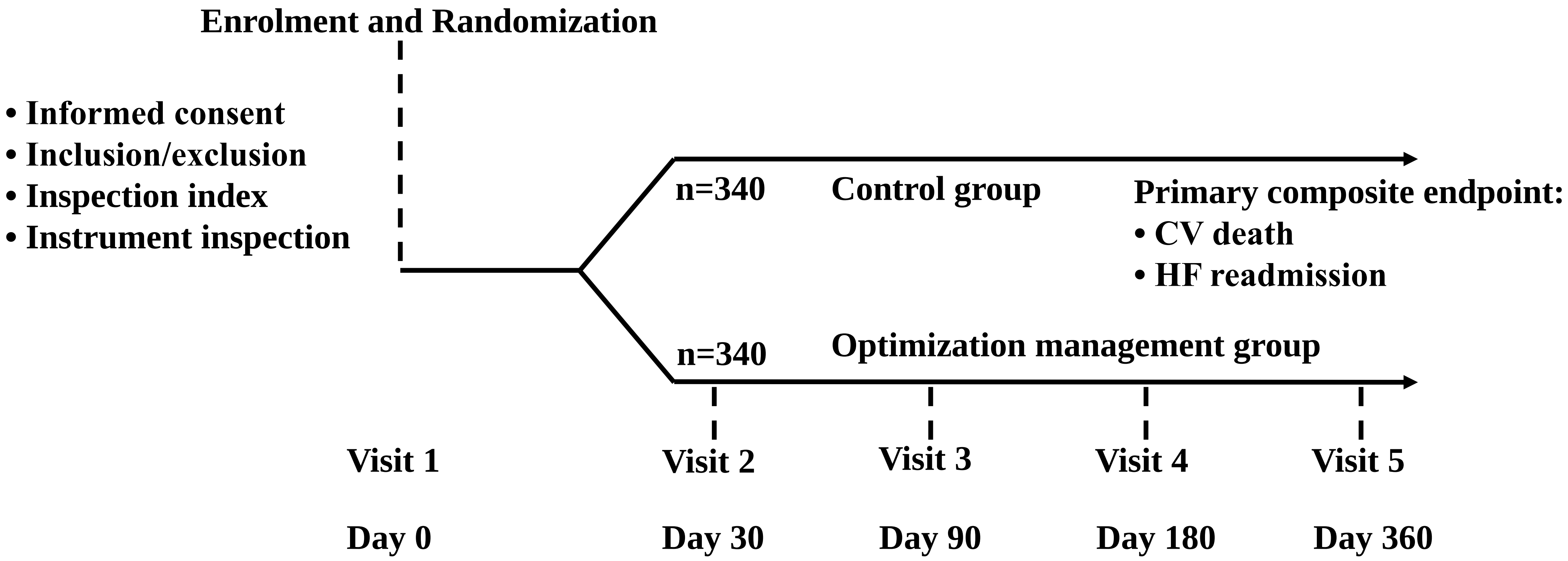
Enrolment and Randomization
• Informed consent
• Inclusion/exclusion
• Inspection index
• Instrument inspection
n=340
Control group
Primary composite endpoint:
• CV death
• HF readmission
n=340
Optimization management group
Visit 1
Visit 2
Visit 3
Visit 4
Visit 5
Day 0
Day 30
Day 90
Day 180
Day 360

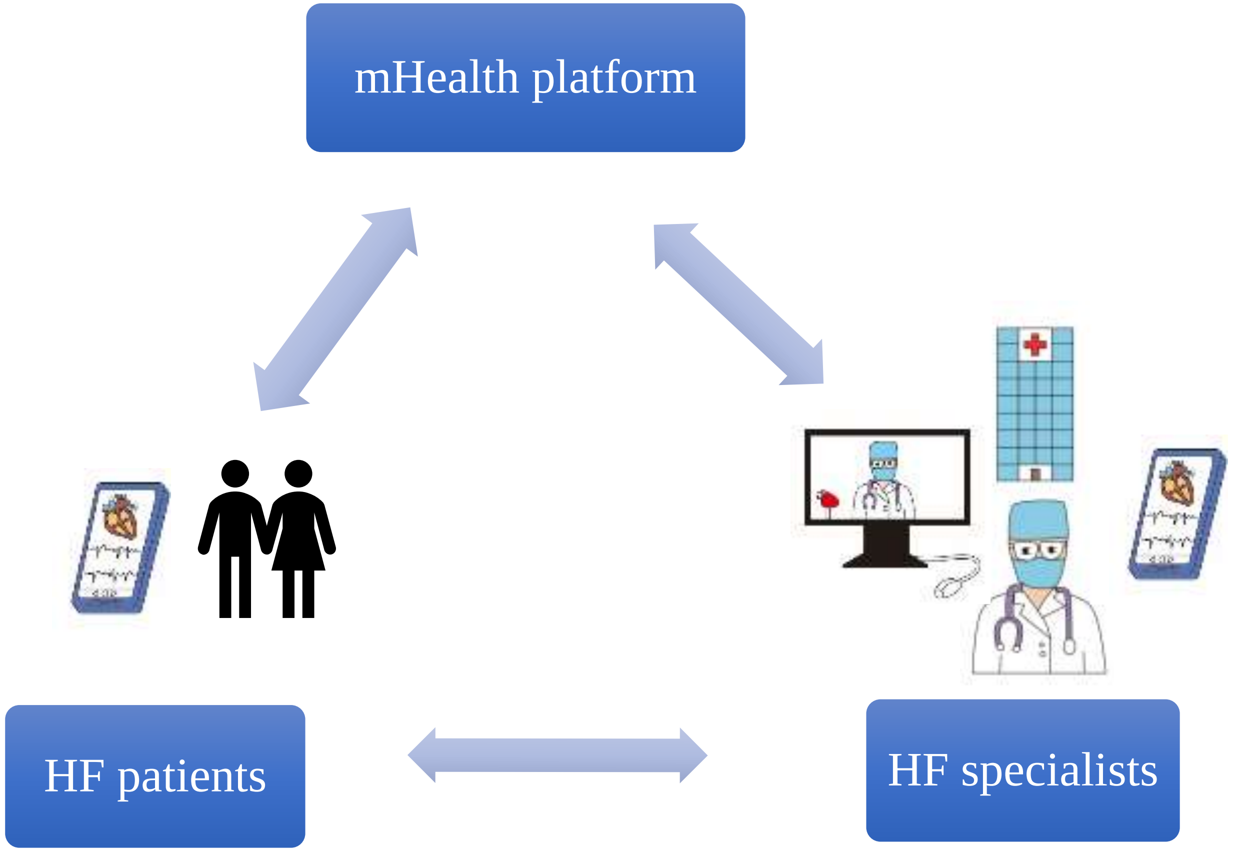
mHealth platform
HF patients
HF specialists

- Willing to provide written informed consent
- 18 years or older
- Left ventricular ejection fraction (LVEF) as measured by the Simpson method needs to be <50% and the New York Heart Association (NYHA) Classes II-IV (NYHA IV in stable phase)
- The patient's brain natriuretic peptide (BNP) concentration should be ≥150 pg/ml or N-terminal pro-B-type natriuretic peptide (NT-proBNP) ≥600 pg/ml, or BNP ≥100 pg/ml (NT-proBNP ≥400 pg/ml) if the patient has been hospitalized for heart failure within 12 months
- Heart failure patients with new or ongoing anti-heart failure medication that has not yet reached target doses and requires adjustment of medication to target or goal measures, or heart failure patients who have been treated to stop the intravenous vasoactive medication and then switched to oral medication after stabilization
- Patients should be proficient in the use of electronic devices

For peer review only - http://bmjopen.bmj.com/site/about/guidelines.xhtml

- Somatic Activity Disorders and Psychiatric Disorders
- Chronic obstructive pulmonary disease (COPD) and Asthma
- Malignant tumor patients (life expectancy < 1 year)
- Plans to receive device or surgical treatment (such as a heart transplant, left ventricular assist device or CRTD implantation and valve replacement surgery during the follow-up period)
- Recurrent episodes of intractable heart failure, Various correctable secondary causes of heart failure (including hyperthyroidism, anemic heart disease, congenital heart disease, organic valvular heart disease)
- Patients with renal failure (creatinine clearance <30 ml/min) or on dialysis treatment
- Hyperkalemia (blood potassium >5.5 mmol/l) at randomization
- Those who are participating in other clinical trials

Reporting checklist for protocol of a clinical trial.

Based on the SPIRIT guidelines.

Instructions to authors

Complete this checklist by entering the page numbers from your manuscript where readers will find each of the items listed below.

Your article may not currently address all the items on the checklist. Please modify your text to include the missing information. If you are certain that an item does not apply, please write "n/a" and provide a short explanation.

Upload your completed checklist as an extra file when you submit to a journal.

In your methods section, say that you used the SPIRITreporting guidelines, and cite them as:

Chan A-W, Tetzlaff JM, Gøtzsche PC, Altman DG, Mann H, Berlin J, Dickersin K, Hróbjartsson A, Schulz KF, Parulekar WR, Krleža-Jerić K, Laupacis A, Moher D. SPIRIT 2013 Explanation and Elaboration: Guidance for protocols of clinical trials. BMJ. 2013;346:e7586

		Reporting Item	Page Number
**Administrative information**			
Title	#1	Descriptive title identifying the study design, population, interventions, and, if applicable, trial acronym	1

For peer review only

Trial registration	#2a	Trial identifier and registry name. If not yet registered, name of intended registry	6
Trial registration: data set	#2b	All items from the World Health Organization Trial Registration Data Set	6
Protocol version	#3	Date and version identifier	n/a
Funding	#4	Sources and types of financial, material, and other support	16
Roles and responsibilities: contributorship	#5a	Names, affiliations, and roles of protocol contributors	1
Roles and responsibilities: sponsor contact information	#5b	Name and contact information for the trial sponsor	1,17
Roles and responsibilities: sponsor and funder	#5c	Role of study sponsor and funders, if any, in study design; collection, management, analysis, and interpretation of data; writing of the report; and the decision to submit the report for publication, including whether they will have ultimate authority over any of these activities	1,17
Roles and responsibilities: committees	#5d	Composition, roles, and responsibilities of the coordinating centre, steering committee, endpoint adjudication committee, data management team, and	13

For peer review only

		other individuals or groups overseeing the trial, if applicable (see Item 21a for data monitoring committee)	
**Introduction**			
Background and rationale	#6a	Description of research question and justification for undertaking the trial, including summary of relevant studies (published and unpublished) examining benefits and harms for each intervention	5,6
Background and rationale: choice of comparators	#6b	Explanation for choice of comparators	4-6
Objectives	#7	Specific objectives or hypotheses	6
Trial design	#8	Description of trial design including type of trial (eg, parallel group, crossover, factorial, single group), allocation ratio, and framework (eg, superiority, equivalence, non-inferiority, exploratory)	7
**Methods: Participants, interventions, and outcomes**			
Study setting	#9	Description of study settings (eg, community clinic, academic hospital) and list of countries where data will be collected. Reference to where list of study sites can be obtained	7

For peer review only

Eligibility criteria	#10	Inclusion and exclusion criteria for participants. If applicable, eligibility criteria for study centres and individuals who will perform the interventions (eg, surgeons, psychotherapists)	8、9
Interventions: description	#11a	Interventions for each group with sufficient detail to allow replication, including how and when they will be administered	7、8
Interventions: modifications	#11b	Criteria for discontinuing or modifying allocated interventions for a given trial participant (eg, drug dose change in response to harms, participant request, or improving / worsening disease)	15
Interventions: adherance	#11c	Strategies to improve adherence to intervention protocols, and any procedures for monitoring adherence (eg, drug tablet return; laboratory tests)	n/a
Interventions: concomitant care	#11d	Relevant concomitant care and interventions that are permitted or prohibited during the trial	n/a
Outcomes	#12	Primary, secondary, and other outcomes, including the specific measurement variable (eg, systolic blood pressure), analysis metric (eg, change from baseline, final value, time to event), method of aggregation (eg, median, proportion), and time point for each outcome. Explanation of the clinical relevance of chosen efficacy and harm outcomes is strongly recommended	10、11

For peer review only

Participant timeline	#13	Time schedule of enrolment, interventions (including any run-ins and washouts), assessments, and visits for participants. A schematic diagram is highly recommended (see Figure)	10
Sample size	#14	Estimated number of participants needed to achieve study objectives and how it was determined, including clinical and statistical assumptions supporting any sample size calculations	11、12
Recruitment	#15	Strategies for achieving adequate participant enrolment to reach target sample size	15,16
**Methods: Assignment of interventions (for controlled trials)**			
Allocation: sequence generation	#16a	Method of generating the allocation sequence (eg, computer-generated random numbers), and list of any factors for stratification. To reduce predictability of a random sequence, details of any planned restriction (eg, blocking) should be provided in a separate document that is unavailable to those who enrol participants or assign interventions	10
Allocation concealment mechanism	#16b	Mechanism of implementing the allocation sequence (eg, central telephone; sequentially numbered, opaque, sealed envelopes), describing any steps to conceal the sequence until interventions are assigned	10

For peer review only

Allocation: implementation	#16c	Who will generate the allocation sequence, who will enrol participants, and who will assign participants to interventions	10
Blinding (masking)	#17a	Who will be blinded after assignment to interventions (eg, trial participants, care providers, outcome assessors, data analysts), and how	6
Blinding (masking): emergency unblinding	#17b	If blinded, circumstances under which unblinding is permissible, and procedure for revealing a participant's allocated intervention during the trial	n/a
**Methods: Data collection, management, and analysis**			
Data collection plan	#18a	Plans for assessment and collection of outcome, baseline, and other trial data, including any related processes to promote data quality (eg, duplicate measurements, training of assessors) and a description of study instruments (eg, questionnaires, laboratory tests) along with their reliability and validity, if known. Reference to where data collection forms can be found, if not in the protocol	10、11
Data collection plan: retention	#18b	Plans to promote participant retention and complete follow-up, including list of any outcome data to be	10、11

For peer review only

		collected for participants who discontinue or deviate from intervention protocols	
Data management	#19	Plans for data entry, coding, security, and storage, including any related processes to promote data quality (eg, double data entry; range checks for data values). Reference to where details of data management procedures can be found, if not in the protocol	10、11
Statistics: outcomes	#20a	Statistical methods for analysing primary and secondary outcomes. Reference to where other details of the statistical analysis plan can be found, if not in the protocol	11、12
Statistics: additional analyses	#20b	Methods for any additional analyses (eg, subgroup and adjusted analyses)	11、12
Statistics: analysis population and missing data	#20c	Definition of analysis population relating to protocol non-adherence (eg, as randomised analysis), and any statistical methods to handle missing data (eg, multiple imputation)	12,13
**Methods: Monitoring**			
Data monitoring: formal committee	#21a	Composition of data monitoring committee (DMC); summary of its role and reporting structure; statement of whether it is independent from the sponsor and competing interests; and reference to where further details about its charter can be found, if not in the	13

For peer review only

		protocol. Alternatively, an explanation of why a DMC is not needed	
Data monitoring: interim analysis	#21b	Description of any interim analyses and stopping guidelines, including who will have access to these interim results and make the final decision to terminate the trial	13
Harms	#22	Plans for collecting, assessing, reporting, and managing solicited and spontaneously reported adverse events and other unintended effects of trial interventions or trial conduct	15
Auditing	#23	Frequency and procedures for auditing trial conduct, if any, and whether the process will be independent from investigators and the sponsor	n/a
**Ethics and dissemination**			
Research ethics approval	#24	Plans for seeking research ethics committee / institutional review board (REC / IRB) approval	15,16
Protocol amendments	#25	Plans for communicating important protocol modifications (eg, changes to eligibility criteria, outcomes, analyses) to relevant parties (eg, investigators, REC / IRBs, trial participants, trial registries, journals, regulators)	n/a

For peer review only

Consent or assent	#26a	Who will obtain informed consent or assent from potential trial participants or authorised surrogates, and how (see Item 32)	9
Consent or assent: ancillary studies	#26b	Additional consent provisions for collection and use of participant data and biological specimens in ancillary studies, if applicable	12
Confidentiality	#27	How personal information about potential and enrolled participants will be collected, shared, and maintained in order to protect confidentiality before, during, and after the trial	10
Declaration of interests	#28	Financial and other competing interests for principal investigators for the overall trial and each study site	17
Data access	#29	Statement of who will have access to the final trial dataset, and disclosure of contractual agreements that limit such access for investigators	n/a
Ancillary and post trial care	#30	Provisions, if any, for ancillary and post-trial care, and for compensation to those who suffer harm from trial participation	15
Dissemination policy: trial results	#31a	Plans for investigators and sponsor to communicate trial results to participants, healthcare professionals, the public, and other relevant groups (eg, via publication, reporting in results databases, or other data sharing arrangements), including any publication restrictions	n/a

For peer review only

Dissemination policy: authorship	#31b	Authorship eligibility guidelines and any intended use of professional writers	1
Dissemination policy: reproducible research	#31c	Plans, if any, for granting public access to the full protocol, participant-level dataset, and statistical code	n/a

Appendices

Informed consent materials	#32	Model consent form and other related documentation given to participants and authorised surrogates	12、13
Biological specimens	#33	Plans for collection, laboratory evaluation, and storage of biological specimens for genetic or molecular analysis in the current trial and for future use in ancillary studies, if applicable	12、13

None The SPIRIT Explanation and Elaboration paper is distributed under the terms of the Creative Commons Attribution License CC-BY-NC. This checklist can be completed online using https://www.goodreports.org/, a tool made by the EQUATOR Network in collaboration with Penelope.ai

For peer review only

BMJ Open

The impHFrEF trial: study protocol for an open-label, multi-center study of improvement the outcome of patients with Heart Failure in China using a mobile hEalth-supported platForm

Journal:	*BMJ Open*
Manuscript ID	bmjopen-2023-081011.R3
Article Type:	Protocol
Date Submitted by the Author:	15-Mar-2024
Complete List of Authors:	Chen, Fuzhong; The First Affiliated Hospital of Nanjing Medical University, Department of Cardiology<br>Li, Guangjuan; The First Affiliated Hospital of Nanjing Medical University, Department of Cardiology; The Friendship Hospital of Ili Kazak Autonomous Prefecture, Department of Cardiology<br>Zhang, Xinxin; Northern Jiangsu People's Hospital, Department of Cardiology<br>Shen, Qin; The First Affiliated Hospital of Nanjing Medical University, Department of Cardiology<br>Wang, Fangfang; Changzhou No.2 People's Hospital, Department of Cardiology<br>Dong, Xiaoyu; The First Affiliated Hospital of Nanjing Medical University, Department of Cardiology<br>Zou, Yu; The First Affiliated Hospital of Nanjing Medical University, Department of Cardiology<br>Chen, Wensen; The First Affiliated Hospital of Nanjing Medical University, Office of Infection Management<br>Xu, Bing; Northern Jiangsu People's Hospital, Department of Cardiology<br>Wang, Junhong; The First Affiliated Hospital of Nanjing Medical University, Department of Cardiology; Jiangsu health administration and development research center
**Primary Subject Heading**:	Cardiovascular medicine
Secondary Subject Heading:	Cardiovascular medicine
Keywords:	Prognosis, Clinical Trial, Heart failure < CARDIOLOGY

SCHOLARONE™
Manuscripts

The impHFrEF trial: study protocol for an open-label, multi-center study of improvement the outcome of patients with Heart Failure in China using a mobile hEalth-supported platForm

Author: Chen Fuzhong[1#], Li Guangjuan[1,2#], Zhang Xinxin[3#], Shen Qin[1], Wang Fangfang[4], Dong Xiaoyu[1], Zou Yu[1], Chen Wensen[5*], Xu Bing[3]*, Wang Junhong[1,6*]

[1]Department of Cardiology, The First Affiliated Hospital of Nanjing Medical University, Nanjing, China.

[2]Department of Cardiology, The Friendship Hospital of Ili Kazak Autonomous Prefecture, Yining, China.

[3]Department of Cardiology, Northern Jiangsu People's Hospital, Yangzhou, China.

[4]Department of Cardiology, Changzhou No.2 People's Hospital, Changzhou, China.

[5]Office of Infection Management, The First Affiliated Hospital of Nanjing Medical University, Nanjing, China.

[6]Jiangsu health administration and development research center, Nanjing, China.

#These authors contributed equally.

* Corresponding to Dr. Chen Wensen: wensenchen@njmu.edu.cn, Dr. Xu Bing, Email: nyxb1980@163.com and Prof. Wang Junhong, E-mail: wangjunhong@jsph.org.cn

For peer review only

Abstract

Background

Patients with chronic heart failure (CHF) often have a long duration of illness, difficulty in attending follow-up visits, and poor adherence to treatment. As a result, they frequently cannot receive guideline-directed medical therapy (GDMT) at the desired or maximum tolerable drug dosage. This leads to high hospitalization and mortality rates for heart failure (HF) patients. Therefore, effective management and monitoring of patients with heart failure to ensure they receive GDMT is crucial for improving their prognosis.

Design and methods

This is a multicenter, open-label, randomized, parallel-group study involving patients with CHF across five centers. The study aims to assess the impact of an optimized guideline-directed medical therapy (GDMT) model for heart failure patients, established on a mobile health (mHealth) platform, compared to a control group. Patients must have a left ventricular ejection fraction (LVEF) of less than 50% and be receiving medication titration therapy that has not yet reached the target dose, with a modest increase in N-terminal pro-B-type natriuretic peptide level. The primary composite outcome is worsening heart failure events (hospitalization or emergency treatment with intravenous fluids) or cardiovascular death.

For peer review only

Ethics and dissemination

On December 22, 2021, this study received ethical approval from the Ethics Review Board of the First Affiliated Hospital of Nanjing Medical University, with the ethics number 2021-SR-530. All study participants will be informed of the research purpose and their participation will be voluntary. Informed consent will be obtained by providing and signing an informed consent form. We will ensure compliance with relevant laws and regulations regarding privacy and data protection. The results of this study will be published in a peer-reviewed academic journal. We will ensure that the dissemination of study results is accurate, clear, and timely.

Keywords

Clinical trial • Heart failure • Optimized management model • moblile health(mHealth) platform

Strengths and limitations

Strengths: (1) The study followed ethical principles and legal regulations, ensuring the safety and legitimacy of the research. (2) As a randomized controlled prospective clinical trial with a large sample size, the study provided reliable results. Limitations: (1) As a prospective clinical study, it is not possible to fully control all variables, which may have an impact on the accuracy of the research results. (2) The generalizability and applicability of the research results may be limited due to variations in medical standards across countries and regions.

For peer review only

Introduction

The term chronic heart failure (CHF) refers to a complex clinical syndrome resulting from abnormal changes in the structure and/or function of the heart owning to numerous factors, leading to impaired ventricular systolic and/or diastolic function [1,2]. The main manifestations are dyspnea, fatigue, and fluid retention (pulmonary circulation congestion, systemic circulation congestion, and peripheral edema) [1,3]. Most patients with acute heart failure (AHF) have partial remission of symptoms after hospitalization and are referred to chronic heart failure; Patients with chronic heart failure often require hospitalization due to acute exacerbation of various triggers. Heart failure has become an important public health issue worldwide due to its high incidence, rehospitalization rate, and death rate [4,5]. China-HF study shows a 4.1% mortality rate for hospitalized patients with heart failure in China [5]. The 2012-2015 Chinese Epidemiological Survey showed that the prevalence of heart failure among adults over 35 years old in China was 1.3% (estimated 13.7 million) [6]. As China's population ages and the incidence of chronic diseases such as coronary heart disease, hypertension, and diabetes increases, improvements in medical care have led to longer survival periods for patients with heart disease, resulting in a continuing trend of increased heart failure prevalence in China.

Guideline-directed medical therapy (GDMT) is a cornerstone of care for patients with CHF. In terms of drug therapy, angiotensin-converting enzyme inhibitors (ACEIs) or angiotensin II receptor antagonists (ARBs), beta-blockers (BBs), and mineralocorticoid

For peer review only

receptor antagonists (MRAs) constitute the standard therapy for the treatment of chronic heart failure. This can significantly improve the clinical symptoms of patients with chronic heart failure and reduce the rate of rehospitalization and mortality. Over the past few years, a variety of new heart failure therapeutic agents have emerged that can improve prognosis. Among them, extensive evidence of the clinical benefit of angiotensin receptor-neprilysin inhibitors (ARNIs) and sodium-glucose co-transporter-2 inhibitors (SGLT2is) has led to a shift from the "ACEI/ARB+BB+MRA" regimen to the " ARNI/ACEI/ARB+SGLT2i+BB+MRA " regimen for improving the prognosis of patients with heart failure with reduced ejection fraction [3,7-9].

Clinical guidelines emphasize the importance of achieving the target dose of guideline-guided medication therapy (GDMT) as much as possible [3,7]. However, due to the long duration of heart failure, it is difficult for patients to be rechecked and examined after discharge due to medical and economic constraints. In addition, severe side effects such as sinus bradycardia, atrioventricular block, and blood pressure reduction can occur during β-blockers, ACEIs/ARBs, and ARNIs therapy, leading to exacerbation and recurrence of heart failure. As a result, patients' adherence to treatment is poor after discharge from the hospital, making it impossible for patients to receive the target dose of medication. This ultimately leads to high rates of rehospitalization and death from heart failure. Recent studies have shown that even in the medically advanced United States, the vast majority of patients with heart failure are not titrated to the target dose or the maximum tolerated dose of BBs and ACEIs/ARBs [10-12]. Therefore, effective

For peer review only

follow-up and management of patients are key to improving their prognosis of patients with heart failure and saving healthcare resources.

Recent studies in China and abroad have attempted to use existing social media tools such as WeChat, Facebook, and Twitter to manage patients with coronary heart disease (CHD) and other cardiovascular diseases to effectively reduce the incidence of cardiovascular events in patients with coronary heart disease. Ge and his team reported in Lancet on the successful use of a WeChat social platform to guide the treatment of patients with CHD through refined management for rehabilitation and secondary prevention [13]. As a result, an APP has been developed for patients with chronic heart failure based on the mobile health(mHealth) platform. The APP can be used to monitor the daily condition of the patient, allowing the physician and patient to communicate in real-time through the software. This establishes a remote monitoring platform with the participation of doctors and patients. By monitoring the arrhythmias of patients with heart failure and their responsiveness to medication, patients can be effectively monitored remotely and guided in the use of medication. Ultimately, patients with heart failure will be effectively managed in a timely and scientific manner, reducing the number of repeated hospitalizations, lowering the death rate of HF patients, and reducing the medical burden on society.

Consequently, the implementation of this study can establish an optimal guideline-directed medical therapy (GDMT) management model for patients with heart failure

based on the mHealth platform. This model can be effectively promoted and popularized, ultimately improving the prognosis of patients with HF and saving social medical resources.

Methods

The study is a multicenter, parallel-group, randomized, open-label, prospective trial in patients with chronic heart failure. The study is scheduled to commence in June 2022 and is expected to conclude in December 2024. The registration number of the study is ChiCTR2200056527.

Study design and conduct

Trial Design Overview

The study is a multicenter, prospective, open-label study. A total of 5 centers participated in this study: Jiangsu Provincial People's Hospital, Northern Jiangsu People's Hospital, the Second Affiliated Hospital of Nanjing Medical University, Changzhou No.2 People’s Hospital, and the Friendship Hospital of Ili Kazak Autonomous Prefecture. In each center, a team of approximately 2-3 clinical physicians is engaged, with the same doctors overseeing both trial and non-trial patients. In addition, a seasoned cardiologist conducts monthly quality control assessments at each center to ensure the rationality of drug treatment adjustments and the integrity of follow-up visits, thus ensuring the homogeneity of the study. A total of 680 patients with CHF are planned to be randomly enrolled and assigned 1:1 to the mhealth

For peer review only

optimized GDMT group (mGDMT group) and the control group, with 340 patients in each group. The population included is heart failure medication not titrated to the target doses or maximum tolerated doses. Patients in the mGDMT group are managed individually and precisely through a mHealth platform application (app), and the dosage of beta-blockers and other HF treatment medications are titrated and adjusted to the patient's tolerated target dose according to the results of the remote mHealth platform. The control group receives the current clinical routine follow-up and treatment. In the control group, patients will be treated by experienced cardiovascular internists at the hospital, who will adjust their treatment plans according to the Chinese guidelines for heart failure. The frequency of clinic visits for these patients will initially be set at 2-4 weeks, but could be extended to once every 1-3 months for those who have reached the target medication dose and have stabilized their condition. Follow-up will be performed at the intervals of 1 month, 3 months, 6 months, and 12 months after enrollment for a total of 12 months to compare clinical symptoms, cardiac function classification, incidence of adverse events, hospitalization rate, mean number of days in hospital, and mortality between the two groups. Figure 1 illustrates the trial procedures of the study.

The mHealth platform App construction

The mHealth platform App builds an interactive platform for doctors and patients to participate together. The App includes a doctor-side and a patient-side portal. The patient side of the software requires the patient to bind to the appropriate doctor and

For peer review only

clock in daily on their weight, urine output, blood pressure, heart rates, heart failure symptoms, and other indicators (laboratory results or examination for local review). The patient-side data is uploaded and presented in a visualized way on the doctor-side. The physician understands the patients' medication reaction based on the data uploaded daily by the patient and communicates effectively with the patient through the App platform dialogue tool. The corresponding physician makes precise and standardized adjustments to the heart failure medication and treatment according to the patients' condition (by utilizing electronic prescriptions). At the same time, the platform sets up an alarm function for extreme values to alert physicians when abnormal values of key indicators appear in patients, facilitating timely adjustment of patients' medication regimens. Throughout the visit, the importance of patient adherence with program requirements regarding daily measurements and symptom reporting is emphasized to ensure timely identification of potential changes in their condition. Figure 2 demonstrates the model for optimal management of patients with heart failure based on a mHealth App.

Patients

The population included in this study is patients with heart failure who have new or ongoing heart failure medication that has not yet reached target doses and requires adjustment of medication to target or goal measures, or patients with heart failure who have been treated to stop the intravenous vasoactive medication and then switched to oral medication after stabilization. The patient's left ventricular ejection fraction (LVEF)

For peer review only

as measured by the Simpson method needs to be <50% and the New York Heart Association (NYHA) Classes II-IV (NYHA Classes IV in stable phase). The patient's brain natriuretic peptide (BNP) concentration should be ≥150 pg/ml or N-terminal pro-B-type natriuretic peptide (NT-proBNP) ≥600 pg/ml, or BNP ≥100 pg/ml if the patient has been hospitalized for heart failure within 12 months (NT-proBNP ≥400 pg/ml). Subjects must meet the age limit of 18 years, be proficient in the use of electronic devices (Proficiency in using WeChat app). Figure 3 displays the inclusion criteria for the trial.

The key exclusion criteria for the impHFrEF trial include Somatic Activity Disorders and Psychiatric Disorders, Chronic obstructive pulmonary disease (COPD) and Asthma, Malignant tumor patients with a life expectancy of less than 1 year, Plans to receive device or surgical treatment such as a heart transplant, left ventricular assist device, CRTD implantation or valve replacement surgery during the follow-up period, Recurrent episodes of intractable heart failure, Various correctable secondary causes of heart failure including hyperthyroidism, anemic heart disease, congenital heart disease, organic valvular heart disease. Patients with renal failure (creatinine clearance <30 ml/min) or on dialysis treatment, hyperkalemia (blood potassium >5.5 mmol/l) at the time of enrollment and those who are participating in other clinical trials are also excluded. Figure 4 presents the exclusion criteria for the trial.

Upon providing their informed consent, eligible patients who consent to participate in this clinical trial will be randomly assigned to their respective groups. All patient data

For peer review only

shall be held in strict confidence and accessible exclusively by authorized personnel. The usage of patients' personal information will be strictly limited to research purposes only. Under no circumstances shall patient information be utilized for any other purposes without explicit written consent from the individuals involved. At the conclusion of the investigation, all patient data will undergo meticulous anonymization procedures, guaranteeing the utmost protection of patient confidentiality.

Randomization

Once patients meet the criteria for randomization, they are assigned by investigators through a central randomization system. Patients in the study are centrally randomized and competitively enrolled in either the mGDMT group or the normal control groups.

Study visits and follow-up

Patients who meet the inclusion criteria will be evaluated upon admission and randomly assigned to either the mGDMT group or the control group after signing an informed consent form. Baseline information, including clinical examination and laboratory measurements, will be collected. Follow-up visits will be conducted on days 30 ± 3, 90 ± 3, 180 ± 3, and 360 ± 3 after randomization. These visits will focus on heart failure symptoms, signs, volume status, and adverse events. ECG, echocardiography, and laboratory results such as troponin, pro-BNP, liver and kidney function will also be reviewed. The schedule for data acquisition is shown in Table 1.

Table 1 Schedule for data acquisition indicated by checkmarks at the specified time point.

Data collected	Baseline	Day	Day	Day	Day

For peer review only

		30	90	180	360
Demographics	✓	N/A[a]	N/A	N/A	N/A
History	✓	N/A	N/A	N/A	N/A
Vital signs	✓	✓	✓	✓	✓
Signs and symptoms	✓	✓	✓	✓	✓
NYHA[b] class	✓	✓	✓	✓	✓
6-min walk test	✓	✓	✓	✓	✓
KCCQ[c] scores	✓	✓	✓	✓	✓
Laboratory results					
BNP[d] levels	✓	N/A	N/A	✓	✓
Troponin	✓	N/A	N/A	✓	✓
Renal function	✓	N/A	N/A	✓	✓
Liver function	✓	N/A	N/A	✓	✓
Examination results					
ECG[e]	✓	N/A	N/A	✓	✓
Echocardiography	✓	N/A	N/A	✓	✓
Endpoints					
Cardiac death	N/A	✓	✓	✓	✓
Number of HF[f]-related hospitalizations since enrollment	N/A	✓	✓	✓	✓
Number of days in the hospital since enrollment	N/A	✓	✓	✓	✓
All-cause death	N/A	✓	✓	✓	✓

[a]N/A: not applicable.

[b]NYHA: New York Heart Association.

[c]KCCQ: Kansas City Cardiomyopathy Questionnaire.

For peer review only

[d]BNP: brain natriuretic peptide.

[e]ECG: Electrocardiogram.

[f]HF: heart failure.

Outcomes

The primary endpoint is a composite endpoint consisting of cardiac death (deaths due to acute myocardial infarction (AMI), HF, systemic embolism, and severe arrhythmic events) and hospitalization due to worsening heart failure symptoms. Worsening heart failure symptoms are defined as the presence of heart failure that cannot be controlled by oral medications and requires hospitalization or emergency room treatment with intravenous medications.

The secondary endpoints primarily include : (i) all-cause death; (ii) cumulative heart failure admissions during the trial; (iii) changes in the 6-minute walk test and heart failure biomarkers; (iv) changes from baseline to 12 months in the total symptom score using the Kansas City Cardiomyopathy Questionnaire (KCCQ) [14].

The study is expected to collect as much clinical information as possible from patients during each visit. This information will be used to compare clinical symptoms, cardiac function class, incidence of adverse events, hospitalization rate, mean days in the hospital, and mortality between the two groups of patients. The efficacy of heart failure medication and adverse events will be monitored and recorded.

For peer review only

Statistical considerations

Sample size assumptions

The incidence rate of endpoint events in the control group was determined based on previous research findings. The annual event rate of heart failure recurrence, cardiogenic and all-cause death in the control group is 15% [15]. The mGDMT group expected an annual event rate of 8% [16]. With a test efficacy of 0.8 and a bilateral alpha of 0.05, a sample size ratio of 1:1 for inclusion of the control and mGDMT groups, and a lost-to-review rate of 5%, a minimum sample size of 340 cases is required for each of the two groups. Therefore, the study expects to include a total of 680 patients.

Methods for statistical analyses

In our experiment, the statistical methods have been meticulously preplanned. All analyses will be conducted using R (version 4.2.2) and SPSS (version 27), both of which are robust and widely accepted statistical software packages. The primary and secondary efficacy analyses will be performed on the full analysis set, adhering to the principle of intention-to-treat. This principle ensures that the results are not biased due to post-randomization exclusions. The full analysis set will include all randomized patients who have provided informed consent and have commenced the indicated treatment.

For data conforming to a normal distribution, it will be expressed as mean ± standard deviation. An independent samples t-test, a parametric test, will be employed for

For peer review only

comparison. For variables with a skewed distribution, they will be expressed as the median and interquartile range (IQR), and a nonparametric test will be used for comparison. This approach allows us to handle data that do not meet the assumptions of parametric tests. Categorical data will be presented as counts and percentages. Chi-square or Fisher exact tests will be utilized for evaluation, depending on the distribution and characteristics of the data. The Kaplan-Meier (K-M) method, a non-parametric statistic used to estimate the survival function from lifetime data, will be used to assess the difference in the incidence of endpoint events between the two groups of patients. In addition, we will conduct a sensitivity analysis to assess the robustness of our findings. A two-sided p-value less than 0.05 will be considered statistically significant. All statistical tests will be two-sided, reflecting our non-directional hypotheses.

Our statistical team, with their extensive experience and expertise, will ensure the accuracy and reliability of our analyses. By adhering to these rigorous statistical methods, we aim to produce precise and trustworthy experimental results.

Ancillary studies

After obtaining informed consent from patients, blood samples will be collected at baseline and during follow-up for the measurement of biomarkers of interest.

Trial design and governance

The study was designed by the Cardiovascular Medicine Department of the First Affiliated Hospital of Nanjing Medical University and reviewed by its Ethics

For peer review only

Committee. The study has completed clinical trial registration and obtained a registration number. There are 5 study centers, and the study will be supervised by the ethics committees of their respective centers during the study implementation.

Patient and Public Involvement

Patients or the public were not involved in the design, or conduct, or reporting, or dissemination plans of our research.

Safety considerations

Patients with heart failure often have a changing condition, with mortality and rehospitalization rates as high as 15% and 30% within 2-3 months of their discharge from the hospital [17]. Therefore, investigators need to evaluate patients' uploaded heart rates, blood pressure, heart failure symptoms, and test results to adjust therapeutic drug doses according to their hemodynamic status and fluid retention. If patients develop renal insufficiency or hyperkalemia during treatment, the medication should also be adjusted immediately according to the guidelines [3]. The trial should be terminated if there is a rapid deterioration in renal function (50% increase in creatinine level or 0.3 mg/dl increase in absolute creatinine level compared to 48 hours before) or severe sinus bradycardia requiring pacing therapy.

Current status

The First Affiliated Hospital of Nanjing Medical University ethically cleared the study

For peer review only

on December 22, 2021, and clinical trial registration was completed on February 7, 2022. Ethics number is 2021-SR-530. The mHealth platform APP was constructed on June 1, 2022. The first subject was recruited and randomized to groups on June 29, 2022. A later phase will rapidly recruit subjects and complete randomization.

Supplementary Information

Ethics and dissemination

On December 22, 2021, this study received ethical approval from the Ethics Review Board of the First Affiliated Hospital of Nanjing Medical University, with the ethics number 2021-SR-530. All study participants will be informed of the research purpose and their participation will be voluntary. Informed consent will be obtained by providing and signing an informed consent form. We will ensure compliance with relevant laws and regulations regarding privacy and data protection. The results of this study will be published in a peer-reviewed academic journal. We will ensure that the dissemination of study results is accurate, clear, and timely.

For peer review only

Funding

This work was supported by the National Natural Science Foundation of China (NSFC 82170269), Jiangsu Province Hospital (the first affiliated hospital with Nanjing Medical University) clinical capacity enhancement project (JSPH-MB-2021-15), the Foundation of Clinical Medical Research Center of Yili Autonomous Prefecture (YL2020ms09), and the open project of Jiangsu health administration and development

research center (JSHD2022009).

Contributorship statement

Chen Fuzhong contributed to the randomization and grouping of patients, as well as the writing and revision of the paper. Li Guangjuan was responsible for patient recruitment and contributed to the paper revision. Zhang Xinxin, Shen Qin, and Wang Fangfang were involved in patient recruitment, data collection, and collation. Dong Xiaoyu assisted in patient randomization, grouping, and data collection. Zou Yu supported data collection and collation. Chen Wensen provided statistical expertise and guidance on trial design. Xu Bing offered guidance on trial design and supervised its implementation. Wang Junhong provided comprehensive guidance on trial design and implementation, oversaw and coordinated the entire research process, and funded the study. Each author played a critical role in ensuring the accuracy and completeness of the research findings and is accountable for the final content of the paper.

Competing Interests Statement

All authors have contributed significantly to the creation of this manuscript and have approved the final version. We affirm that there are no competing interests, financial or otherwise, that could influence the interpretation or presentation of the results presented in this work.

References

1. Guan X, Sun Z. The Role of Intestinal Flora and Its Metabolites in Heart Failure.

For peer review only

Infection and drug resistance 2023;16:51-64. (In eng). DOI: 10.2147/idr.S390582.

2. Kanodia J, Lo A, Baldwin RM, Colley K, Zhou K, Bourdet DL. Safety, Pharmacokinetics, and Pharmacodynamics of TD-0714, a Novel Potent Neprilysin Inhibitor in Healthy Adult and Elderly Subjects. Clinical and translational science 2020;13(6):1307-1315. (In eng). DOI: 10.1111/cts.12831.

3. McDonagh TA, Metra M, Adamo M, et al. 2021 ESC Guidelines for the diagnosis and treatment of acute and chronic heart failure. European heart journal 2021;42(36):3599-3726. (In eng). DOI: 10.1093/eurheartj/ehab368.

4. Chow SL, Maisel AS, Anand I, et al. Role of Biomarkers for the Prevention, Assessment, and Management of Heart Failure: A Scientific Statement From the American Heart Association. Circulation 2017;135(22):e1054-e1091. (In eng). DOI: 10.1161/cir.0000000000000490.

5. Zhang Y, Zhang J, Butler J, et al. Contemporary Epidemiology, Management, and Outcomes of Patients Hospitalized for Heart Failure in China: Results From the China Heart Failure (China-HF) Registry. Journal of cardiac failure 2017;23(12):868-875. (In eng). DOI: 10.1016/j.cardfail.2017.09.014.

6. Hao G, Wang X, Chen Z, et al. Prevalence of heart failure and left ventricular dysfunction in China: the China Hypertension Survey, 2012-2015. European journal of heart failure 2019;21(11):1329-1337. (In eng). DOI: 10.1002/ejhf.1629.

7. Heidenreich PA, Bozkurt B, Aguilar D, et al. 2022 AHA/ACC/HFSA Guideline for the Management of Heart Failure: Executive Summary: A Report of the American College of Cardiology/American Heart Association Joint Committee on Clinical Practice

For peer review only

Guidelines. Circulation 2022;145(18):e876-e894. (In eng). DOI: 10.1161/cir.0000000000001062.

8. Maddox TM, Januzzi JL, Jr., Allen LA, et al. 2021 Update to the 2017 ACC Expert Consensus Decision Pathway for Optimization of Heart Failure Treatment: Answers to 10 Pivotal Issues About Heart Failure With Reduced Ejection Fraction: A Report of the American College of Cardiology Solution Set Oversight Committee. Journal of the American College of Cardiology 2021;77(6):772-810. (In eng). DOI: 10.1016/j.jacc.2020.11.022.

9. McMurray JJV, Packer M. How Should We Sequence the Treatments for Heart Failure and a Reduced Ejection Fraction?: A Redefinition of Evidence-Based Medicine. Circulation 2021;143(9):875-877. (In eng). DOI: 10.1161/circulationaha.120.052926.

10. Chang HY, Wang CC, Wei J, et al. Gap between guidelines and clinical practice in heart failure with reduced ejection fraction: Results from TSOC-HFrEF registry. Journal of the Chinese Medical Association : JCMA 2017;80(12):750-757. (In eng). DOI: 10.1016/j.jcma.2017.04.011.

11. Greene SJ, Fonarow GC, DeVore AD, et al. Titration of Medical Therapy for Heart Failure With Reduced Ejection Fraction. Journal of the American College of Cardiology 2019;73(19):2365-2383. (In eng). DOI: 10.1016/j.jacc.2019.02.015.

12. Jungbauer CG, Maier LS, Emoto K, Zirille FM, Mirro MJ. Achieving Guideline-Directed Heart Rate ControlEarly Posthospitalization. The American journal of cardiology 2019;123(7):1096-1100. (In eng). DOI: 10.1016/j.amjcard.2018.12.032.

13. Dorje T, Zhao G, Tso K, et al. Smartphone and social media-based cardiac

For peer review only

rehabilitation and secondary prevention in China (SMART-CR/SP): a parallel-group, single-blind, randomised controlled trial. The Lancet Digital health 2019;1(7):e363-e374. (In eng). DOI: 10.1016/s2589-7500(19)30151-7.

14. Green CP, Porter CB, Bresnahan DR, Spertus JA. Development and evaluation of the Kansas City Cardiomyopathy Questionnaire: a new health status measure for heart failure. Journal of the American College of Cardiology 2000;35(5):1245-55. (In eng). DOI: 10.1016/s0735-1097(00)00531-3.

15. McMurray JJ, Packer M, Desai AS, et al. Angiotensin-neprilysin inhibition versus enalapril in heart failure. The New England journal of medicine 2014;371(11):993-1004. (In eng). DOI: 10.1056/NEJMoa1409077.

16. Kotb A, Cameron C, Hsieh S, Wells G. Comparative effectiveness of different forms of telemedicine for individuals with heart failure (HF): a systematic review and network meta-analysis. PloS one 2015;10(2):e0118681. (In eng). DOI: 10.1371/journal.pone.0118681.

17. Greene SJ, Fonarow GC, Vaduganathan M, Khan SS, Butler J, Gheorghiade M. The vulnerable phase after hospitalization for heart failure. Nature reviews Cardiology 2015;12(4):220-9. (In eng). DOI: 10.1038/nrcardio.2015.14.

Figure 1 Trial design. CV, cardiovascular; HF, heart failure.

For peer review only

Figure 2 Optimized GDMT model for managing HF patients based on the mHealth platform. mHealth, mobile health; HF, heart failure; GDMT, guideline directed medical therapy.

Figure 3 Patient inclusion criteria.
Figure 4 Patient exclusion criteria.

For peer review only

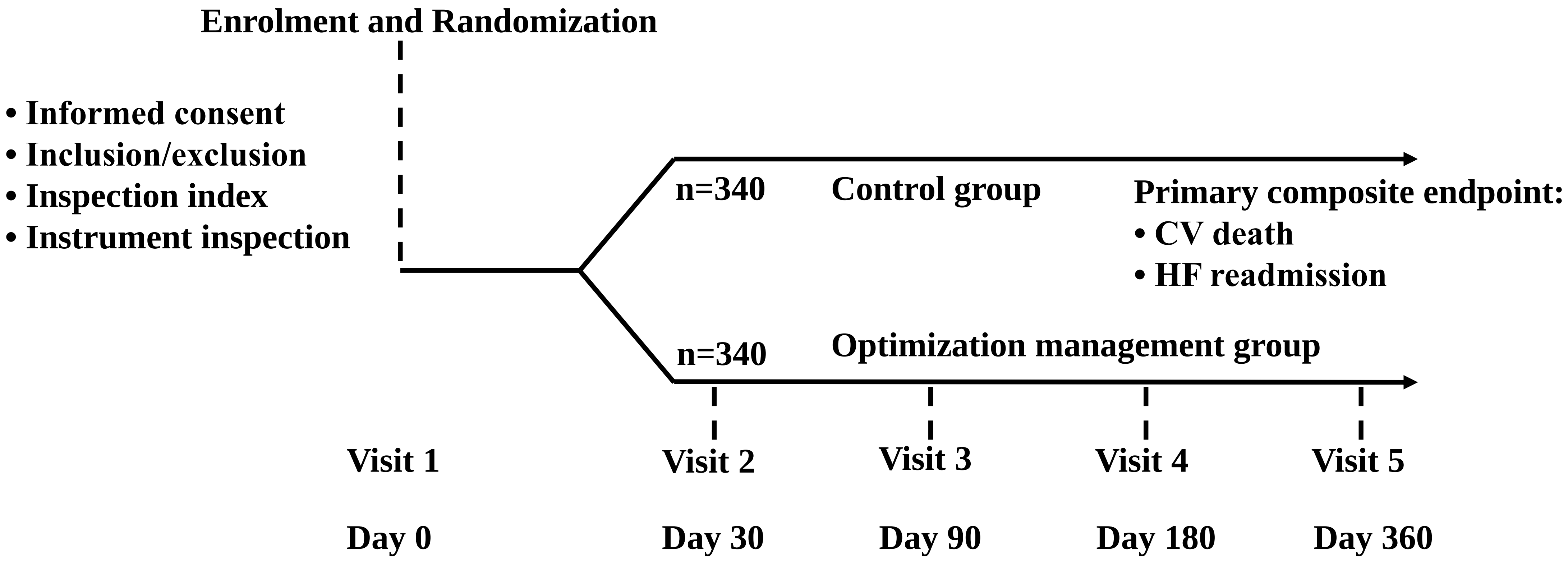
Enrolment and Randomization
• Informed consent
• Inclusion/exclusion
• Inspection index
• Instrument inspection
n=340
Control group
Primary composite endpoint:
• CV death
• HF readmission
n=340
Optimization management group
Visit 1
Day 0
Visit 2
Day 30
Visit 3
Day 90
Visit 4
Day 180
Visit 5
Day 360

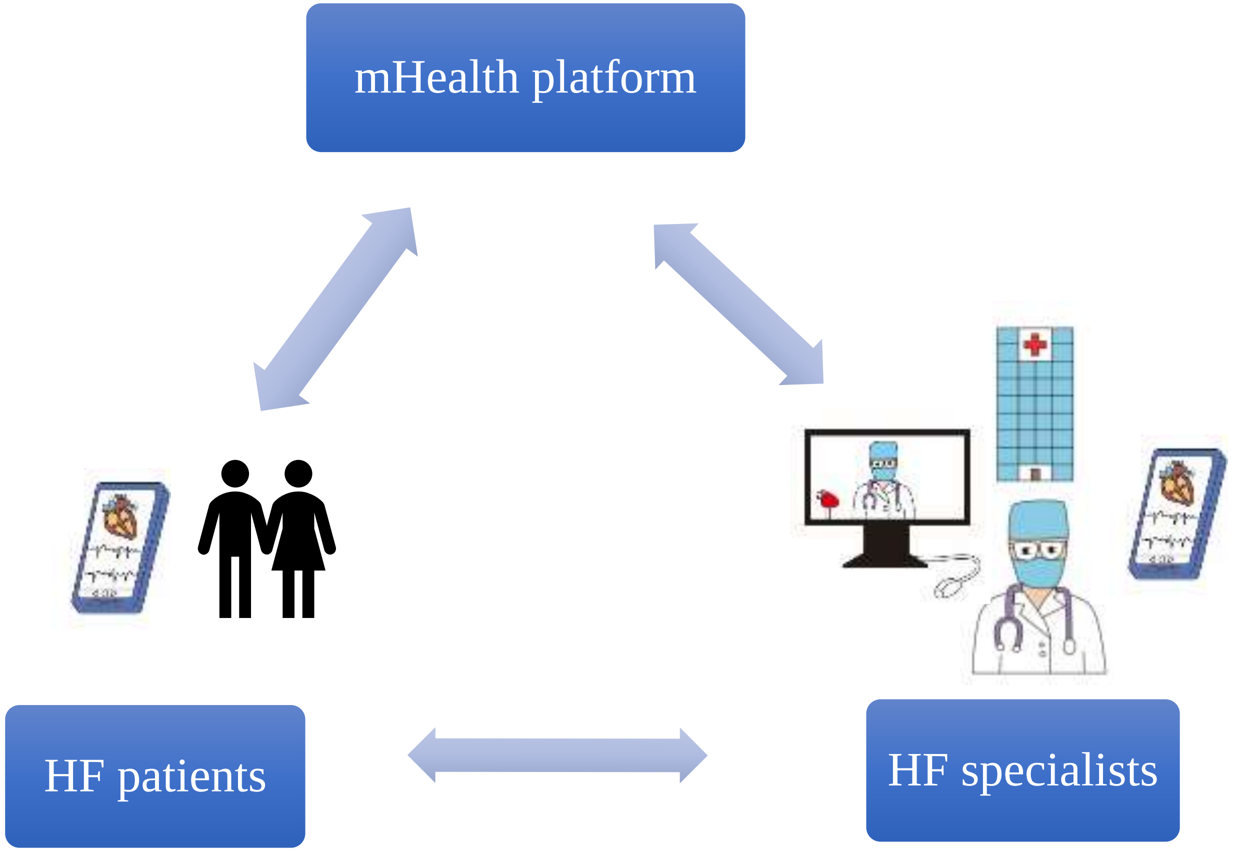
mHealth platform
HF patients
HF specialists

- Willing to provide written informed consent
- 18 years or older
- Left ventricular ejection fraction (LVEF) as measured by the Simpson method needs to be <50% and the New York Heart Association (NYHA) Classes II-IV (NYHA IV in stable phase)
- The patient's brain natriuretic peptide (BNP) concentration should be ≥150 pg/ml or N-terminal pro-B-type natriuretic peptide (NT-proBNP) ≥600 pg/ml, or BNP ≥100 pg/ml (NT-proBNP ≥400 pg/ml) if the patient has been hospitalized for heart failure within 12 months
- Heart failure patients with new or ongoing anti-heart failure medication that has not yet reached target doses and requires adjustment of medication to target or goal measures, or heart failure patients who have been treated to stop the intravenous vasoactive medication and then switched to oral medication after stabilization
- Patients should be proficient in the use of electronic devices

- Somatic Activity Disorders and Psychiatric Disorders
- Chronic obstructive pulmonary disease (COPD) and Asthma
- Malignant tumor patients (life expectancy < 1 year)
- Plans to receive device or surgical treatment (such as a heart transplant, left ventricular assist device or CRTD implantation and valve replacement surgery during the follow-up period)
- Recurrent episodes of intractable heart failure, Various correctable secondary causes of heart failure (including hyperthyroidism, anemic heart disease, congenital heart disease, organic valvular heart disease)
- Patients with renal failure (creatinine clearance <30 ml/min) or on dialysis treatment
- Hyperkalemia (blood potassium >5.5 mmol/l) at randomization
- Those who are participating in other clinical trials

For peer review only - http://bmjopen.bmj.com/site/about/guidelines.xhtml

Reporting checklist for protocol of a clinical trial.

Based on the SPIRIT guidelines.

Instructions to authors

Complete this checklist by entering the page numbers from your manuscript where readers will find each of the items listed below.

Your article may not currently address all the items on the checklist. Please modify your text to include the missing information. If you are certain that an item does not apply, please write "n/a" and provide a short explanation.

Upload your completed checklist as an extra file when you submit to a journal.

In your methods section, say that you used the SPIRITreporting guidelines, and cite them as:

Chan A-W, Tetzlaff JM, Gøtzsche PC, Altman DG, Mann H, Berlin J, Dickersin K, Hróbjartsson A, Schulz KF, Parulekar WR, Krleža-Jerić K, Laupacis A, Moher D. SPIRIT 2013 Explanation and Elaboration: Guidance for protocols of clinical trials. BMJ. 2013;346:e7586

		Reporting Item	Page Number
**Administrative information**			
Title	#1	Descriptive title identifying the study design, population, interventions, and, if applicable, trial acronym	1

For peer review only

Trial registration	#2a	Trial identifier and registry name. If not yet registered, name of intended registry	6
Trial registration: data set	#2b	All items from the World Health Organization Trial Registration Data Set	6
Protocol version	#3	Date and version identifier	n/a
Funding	#4	Sources and types of financial, material, and other support	16
Roles and responsibilities: contributorship	#5a	Names, affiliations, and roles of protocol contributors	1
Roles and responsibilities: sponsor contact information	#5b	Name and contact information for the trial sponsor	1,17
Roles and responsibilities: sponsor and funder	#5c	Role of study sponsor and funders, if any, in study design; collection, management, analysis, and interpretation of data; writing of the report; and the decision to submit the report for publication, including whether they will have ultimate authority over any of these activities	1,17
Roles and responsibilities: committees	#5d	Composition, roles, and responsibilities of the coordinating centre, steering committee, endpoint adjudication committee, data management team, and	13

For peer review only

		other individuals or groups overseeing the trial, if applicable (see Item 21a for data monitoring committee)	
**Introduction**			
Background and rationale	#6a	Description of research question and justification for undertaking the trial, including summary of relevant studies (published and unpublished) examining benefits and harms for each intervention	5,6
Background and rationale: choice of comparators	#6b	Explanation for choice of comparators	4-6
Objectives	#7	Specific objectives or hypotheses	6
Trial design	#8	Description of trial design including type of trial (eg, parallel group, crossover, factorial, single group), allocation ratio, and framework (eg, superiority, equivalence, non-inferiority, exploratory)	7
**Methods: Participants, interventions, and outcomes**			
Study setting	#9	Description of study settings (eg, community clinic, academic hospital) and list of countries where data will be collected. Reference to where list of study sites can be obtained	7

For peer review only

Eligibility criteria	#10	Inclusion and exclusion criteria for participants. If applicable, eligibility criteria for study centres and individuals who will perform the interventions (eg, surgeons, psychotherapists)	8、9
Interventions: description	#11a	Interventions for each group with sufficient detail to allow replication, including how and when they will be administered	7、8
Interventions: modifications	#11b	Criteria for discontinuing or modifying allocated interventions for a given trial participant (eg, drug dose change in response to harms, participant request, or improving / worsening disease)	15
Interventions: adherance	#11c	Strategies to improve adherence to intervention protocols, and any procedures for monitoring adherence (eg, drug tablet return; laboratory tests)	n/a
Interventions: concomitant care	#11d	Relevant concomitant care and interventions that are permitted or prohibited during the trial	n/a
Outcomes	#12	Primary, secondary, and other outcomes, including the specific measurement variable (eg, systolic blood pressure), analysis metric (eg, change from baseline, final value, time to event), method of aggregation (eg, median, proportion), and time point for each outcome. Explanation of the clinical relevance of chosen efficacy and harm outcomes is strongly recommended	10、11

For peer review only

Participant timeline	#13	Time schedule of enrolment, interventions (including any run-ins and washouts), assessments, and visits for participants. A schematic diagram is highly recommended (see Figure)	10
Sample size	#14	Estimated number of participants needed to achieve study objectives and how it was determined, including clinical and statistical assumptions supporting any sample size calculations	11、12
Recruitment	#15	Strategies for achieving adequate participant enrolment to reach target sample size	15,16
**Methods: Assignment of interventions (for controlled trials)**			
Allocation: sequence generation	#16a	Method of generating the allocation sequence (eg, computer-generated random numbers), and list of any factors for stratification. To reduce predictability of a random sequence, details of any planned restriction (eg, blocking) should be provided in a separate document that is unavailable to those who enrol participants or assign interventions	10
Allocation concealment mechanism	#16b	Mechanism of implementing the allocation sequence (eg, central telephone; sequentially numbered, opaque, sealed envelopes), describing any steps to conceal the sequence until interventions are assigned	10

For peer review only

Allocation: implementation	#16c	Who will generate the allocation sequence, who will enrol participants, and who will assign participants to interventions	10
Blinding (masking)	#17a	Who will be blinded after assignment to interventions (eg, trial participants, care providers, outcome assessors, data analysts), and how	6
Blinding (masking): emergency unblinding	#17b	If blinded, circumstances under which unblinding is permissible, and procedure for revealing a participant's allocated intervention during the trial	n/a
**Methods: Data collection, management, and analysis**			
Data collection plan	#18a	Plans for assessment and collection of outcome, baseline, and other trial data, including any related processes to promote data quality (eg, duplicate measurements, training of assessors) and a description of study instruments (eg, questionnaires, laboratory tests) along with their reliability and validity, if known. Reference to where data collection forms can be found, if not in the protocol	10、11
Data collection plan: retention	#18b	Plans to promote participant retention and complete follow-up, including list of any outcome data to be	10、11

For peer review only

		collected for participants who discontinue or deviate from intervention protocols	
Data management	#19	Plans for data entry, coding, security, and storage, including any related processes to promote data quality (eg, double data entry; range checks for data values). Reference to where details of data management procedures can be found, if not in the protocol	10、11
Statistics: outcomes	#20a	Statistical methods for analysing primary and secondary outcomes. Reference to where other details of the statistical analysis plan can be found, if not in the protocol	11、12
Statistics: additional analyses	#20b	Methods for any additional analyses (eg, subgroup and adjusted analyses)	11、12
Statistics: analysis population and missing data	#20c	Definition of analysis population relating to protocol non-adherence (eg, as randomised analysis), and any statistical methods to handle missing data (eg, multiple imputation)	12,13
**Methods: Monitoring**			
Data monitoring: formal committee	#21a	Composition of data monitoring committee (DMC); summary of its role and reporting structure; statement of whether it is independent from the sponsor and competing interests; and reference to where further details about its charter can be found, if not in the	13

For peer review only

		protocol. Alternatively, an explanation of why a DMC is not needed	
Data monitoring: interim analysis	#21b	Description of any interim analyses and stopping guidelines, including who will have access to these interim results and make the final decision to terminate the trial	13
Harms	#22	Plans for collecting, assessing, reporting, and managing solicited and spontaneously reported adverse events and other unintended effects of trial interventions or trial conduct	15
Auditing	#23	Frequency and procedures for auditing trial conduct, if any, and whether the process will be independent from investigators and the sponsor	n/a
**Ethics and dissemination**			
Research ethics approval	#24	Plans for seeking research ethics committee / institutional review board (REC / IRB) approval	15,16
Protocol amendments	#25	Plans for communicating important protocol modifications (eg, changes to eligibility criteria, outcomes, analyses) to relevant parties (eg, investigators, REC / IRBs, trial participants, trial registries, journals, regulators)	n/a

For peer review only

Consent or assent	#26a	Who will obtain informed consent or assent from potential trial participants or authorised surrogates, and how (see Item 32)	9
Consent or assent: ancillary studies	#26b	Additional consent provisions for collection and use of participant data and biological specimens in ancillary studies, if applicable	12
Confidentiality	#27	How personal information about potential and enrolled participants will be collected, shared, and maintained in order to protect confidentiality before, during, and after the trial	10
Declaration of interests	#28	Financial and other competing interests for principal investigators for the overall trial and each study site	17
Data access	#29	Statement of who will have access to the final trial dataset, and disclosure of contractual agreements that limit such access for investigators	n/a
Ancillary and post trial care	#30	Provisions, if any, for ancillary and post-trial care, and for compensation to those who suffer harm from trial participation	15
Dissemination policy: trial results	#31a	Plans for investigators and sponsor to communicate trial results to participants, healthcare professionals, the public, and other relevant groups (eg, via publication, reporting in results databases, or other data sharing arrangements), including any publication restrictions	n/a

For peer review only

Dissemination policy: authorship	#31b	Authorship eligibility guidelines and any intended use of professional writers	1
Dissemination policy: reproducible research	#31c	Plans, if any, for granting public access to the full protocol, participant-level dataset, and statistical code	n/a

Appendices

Informed consent materials	#32	Model consent form and other related documentation given to participants and authorised surrogates	12、13
Biological specimens	#33	Plans for collection, laboratory evaluation, and storage of biological specimens for genetic or molecular analysis in the current trial and for future use in ancillary studies, if applicable	12、13

None The SPIRIT Explanation and Elaboration paper is distributed under the terms of the Creative Commons Attribution License CC-BY-NC. This checklist can be completed online using https://www.goodreports.org/, a tool made by the EQUATOR Network in collaboration with Penelope.ai

For peer review only
